# Effectiveness of universal school-based mindfulness training compared with normal school provision on teacher mental health and school climate: results of the MYRIAD cluster randomised controlled trial

**DOI:** 10.1136/ebmental-2022-300424

**Published:** 2022-07-07

**Authors:** Willem Kuyken, Susan Ball, Catherine Crane, Poushali Ganguli, Benjamin Jones, Jesus Montero-Marin, Elizabeth Nuthall, Anam Raja, Laura Taylor, Kate Tudor, Russell M Viner, Matthew Allwood, Louise Aukland, Darren Dunning, Tríona Casey, Nicola Dalrymple, Katherine De Wilde, Eleanor-Rose Farley, Jennifer Harper, Verena Hinze, Nils Kappelmann, Maria Kempnich, Liz Lord, Emma Medlicott, Lucy Palmer, Ariane Petit, Alice Philips, Isobel Pryor-Nitsch, Lucy Radley, Anna Sonley, Jem Shackleford, Alice Tickell, MYRIAD Team, Sarah-Jayne Blakemore, Obioha C Ukoumunne, Mark T Greenberg, Tamsin Ford, Tim Dalgleish, Sarah Byford, J Mark G Williams

**Affiliations:** 1 Department of Psychiatry, Warneford Hospital, University of Oxford, Oxford, UK; 2 NIHR Applied Research Collaboration Southwest Peninsula (PenARC), University of Exeter, Exeter, Devon, UK; 3 King’s College London, King’s Health Economics, Institute of Psychiatry, Psychology and Neuroscience, De Crespigny Park, London, UK; 4 Teaching, Research and Innovation Unit, Parc Sanitari Sant Joan de Déu, Sant Boi de Llobregat, Spain; 5 Population, Policy & Practice research programme, UCL Great Ormond St. Institute of Child Health, London, UK; 6 Medical Research Council Cognition and Brain Sciences Unit, University of Cambridge, Cambridge, UK; 7 Department of Psychology, University of Cambridge, Cambridge, UK; 8 UCL Institute of Cognitive Neuroscience, London, UK; 9 Department of Human Development and Family Studies, Pennsylvania State University, Pennsylvania, Pennsylvania, USA; 10 Department of Psychiatry, University of Cambridge, Cambridge Biomedical Campus, Cambridge, UK

**Keywords:** Child & adolescent psychiatry, Depression & mood disorders

## Abstract

**Background:**

Education is broader than academic teaching. It includes teaching students social–emotional skills both directly and indirectly through a positive school climate.

**Objective:**

To evaluate if a universal school-based mindfulness training (SBMT) enhances teacher mental health and school climate.

**Methods:**

The My Resilience in Adolescence parallel group, cluster randomised controlled trial (registration: ISRCTN86619085; funding: Wellcome Trust (WT104908/Z/14/Z, WT107496/Z/15/Z)) recruited 85 schools (679 teachers) delivering social and emotional teaching across the UK. Schools (clusters) were randomised 1:1 to either continue this provision (teaching as usual (TAU)) or include universal SBMT. Data on teacher mental health and school climate were collected at prerandomisation, postpersonal mindfulness and SBMT teacher training, after delivering SBMT to students, and at 1-year follow-up.

**Finding:**

Schools were recruited in academic years 2016/2017 and 2017/2018. Primary analysis (SBMT: 43 schools/362 teachers; TAU: 41 schools/310 teachers) showed that after delivering SBMT to students, SBMT versus TAU enhanced teachers’ mental health (burnout) and school climate. Adjusted standardised mean differences (SBMT minus TAU) were: exhaustion (−0.22; 95% CI −0.38 to −0.05); personal accomplishment (−0.21; −0.41, −0.02); school leadership (0.24; 0.04, 0.44); and respectful climate (0.26; 0.06, 0.47). Effects on burnout were not significant at 1-year follow-up. Effects on school climate were maintained only for respectful climate. No SBMT-related serious adverse events were reported.

**Conclusions:**

SBMT supports short-term changes in teacher burnout and school climate. Further work is required to explore how best to sustain improvements.

**Clinical implications:**

SBMT has limited effects on teachers’ mental and school climate. Innovative approaches to support and preserve teachers’ mental health and school climate are needed.

**Key messages**

**What is already known on this topic**

- There are meta-analyses demonstrating the effectiveness of mindfulness-based programmes (MBPs) on mental health in a range of populations and contexts, including school teachers in the educational sector. However, these studies are compromised by sample size and design, including inadequate consideration of the MBP curriculum, teacher engagement, outcome assessment over longer periods and publication bias, nor effects on wider school climate.

**What this study adds**

- This study included careful consideration of the MBP itself, fidelity, teacher engagement, follow-up at 1 year, as well as measures of school climate. It suggests that school-based mindfulness training reduced teachers’ burnout and enhanced some dimensions of school climate, but that these effects largely wash out over time.

**How this study might affect research, practice or policy**

- MBPs can improve teacher’s mental health in the short term and may also improve school climate. The next generation of research needs to consider how best to support teacher mental health and school climate in sustainable ways and what role MBP might play in this by paying close attention to contextual and implementation factors.

## Background

There is a growing consensus that the purpose of schools extends beyond academic teaching, to a social–emotional education that prepares young people for life.^1–3^ Schools can develop young people’s social–emotional–behavioural competencies directly through teaching social–emotional learning (SEL) curricula,^4^ and indirectly through a positive school climate.^5^ School climate is a broad multidimensional term that includes perceptions of different aspects of the school experience in terms of academic, community, safety and structural features. It refers to all aspects of school life, for example, leadership, teaching/learning, professional development, partnership, relationships, connectedness, respect, social/emotional/physical safety, discipline, the school environment, organisation and facilities.^5 6^ There is promising evidence that a more positive school climate is associated with students’ improved mental and physical health, less absenteeism, better behaviour, less substance use and better academic outcomes.^5 7^

Teaching can be both rewarding and stressful, as many aspects of teaching are demanding: workload, inspections, high expectations from school senior leadership and parents, students’ behavioural/emotional problems, societal issues that inevitably affect schools (eg, deprivation, discrimination, inequality) and changing educational policies and priorities.^8^ As a consequence, teacher stress, burnout and absenteeism are common, and there is a high turnover in the teaching profession.^9^ As such, there are good reasons for schools to safeguard and promote teachers’ mental health.

SEL programmes are focused on helping individuals manage their emotional states, reach goals with empathy for others, maintain positive relationships and make responsible decisions.^4^ These programmes can enhance both school climate and teachers’ mental health in a range of ways, for example, effective SEL can promote better behaviour among students and effective classroom management, supporting teachers’ self-efficacy, which in turn improves stress, job satisfaction and retention.^10–13^

One approach to SEL that has shown promise is mindfulness training (MT). School-based mindfulness training (SBMT) delivered by teachers typically involves teachers learning mindfulness themselves, followed by training in how to deliver MT to students, before going on to deliver MT to students. MT involves learning attentional control, and emotional and social regulation that can enhance resilience in the face of stressors, and promote mental health. Systematic reviews suggest positive effects of MT on both young people’s^14^ and teachers’ mental health.^15^ Most studies are preliminary and of inconsistent quality, especially with regards to lack of statistical power, unclear specification of the intervention, inadequate or no measurement of intervention fidelity, weak choice of comparator and short follow-up. In addition, there is almost no research on the effects of SBMT on school climate.^16^

This study is part of a larger programme of work examining the effectiveness, cost-effectiveness, mechanisms, scalability and implementation of SBMT (‘MYRIAD: My Resilience in Adolescence’).^17–19^

## Objective

This paper addresses the MYRIAD trial’s secondary question: Is universal SBMT more effective than teaching as usual (TAU) in supporting teachers’ mental health and school climate? An ancillary aim was to examine teachers’ engagement with their personal MT and with SBMT, and whether this engagement was associated with teachers’ mental health and school climate.

## Method

The trial was reported in accordance with the Consolidated Standards of Reporting Trials guidelines for cluster randomised controlled trials (RCTs).^20^ The study design and procedures are presented in full in the published trial protocol^17^ and update describing study enhancements and adaptations.^18^

### Study design and participants

We used a cluster RCT design, with schools (clusters) as the unit of randomisation, comparing SBMT and TAU, with regard to teachers’ mental health and school climate. All mainstream schools (ages 11–16) in the four UK regions (England, Northern Ireland, Scotland and Wales) were eligible if they had a substantive appointed headteacher, had not been judged inadequate in their most recent official inspection (to mitigate any difficulties in trial implementation) and had a strategy/structure in place for delivery of adequate SEL curricula. Consent was sought at the school level by the corresponding headteacher and by all involved teachers. Both were obtained before randomisation. We recruited schools (n=85) in the academic years 2016/2017 (cohort 1; K=13) and 2017/2018 (cohort 2; K=72).

Prior to randomisation, all study schools nominated up to 15 teachers, normally on substantive contracts to minimise attrition. The headteachers agreed to enable their nominated teachers to participate in the research, complete their personal MT and SBMT teacher training and deliver the SBMT curriculum to students should their school be randomised to the intervention arm. Although up to 15 teachers started the personal MT, a maximum of 5 could go on to training in the SBMT curriculum, and only a subset of those would actually deliver it to students.

**Figure 1:**
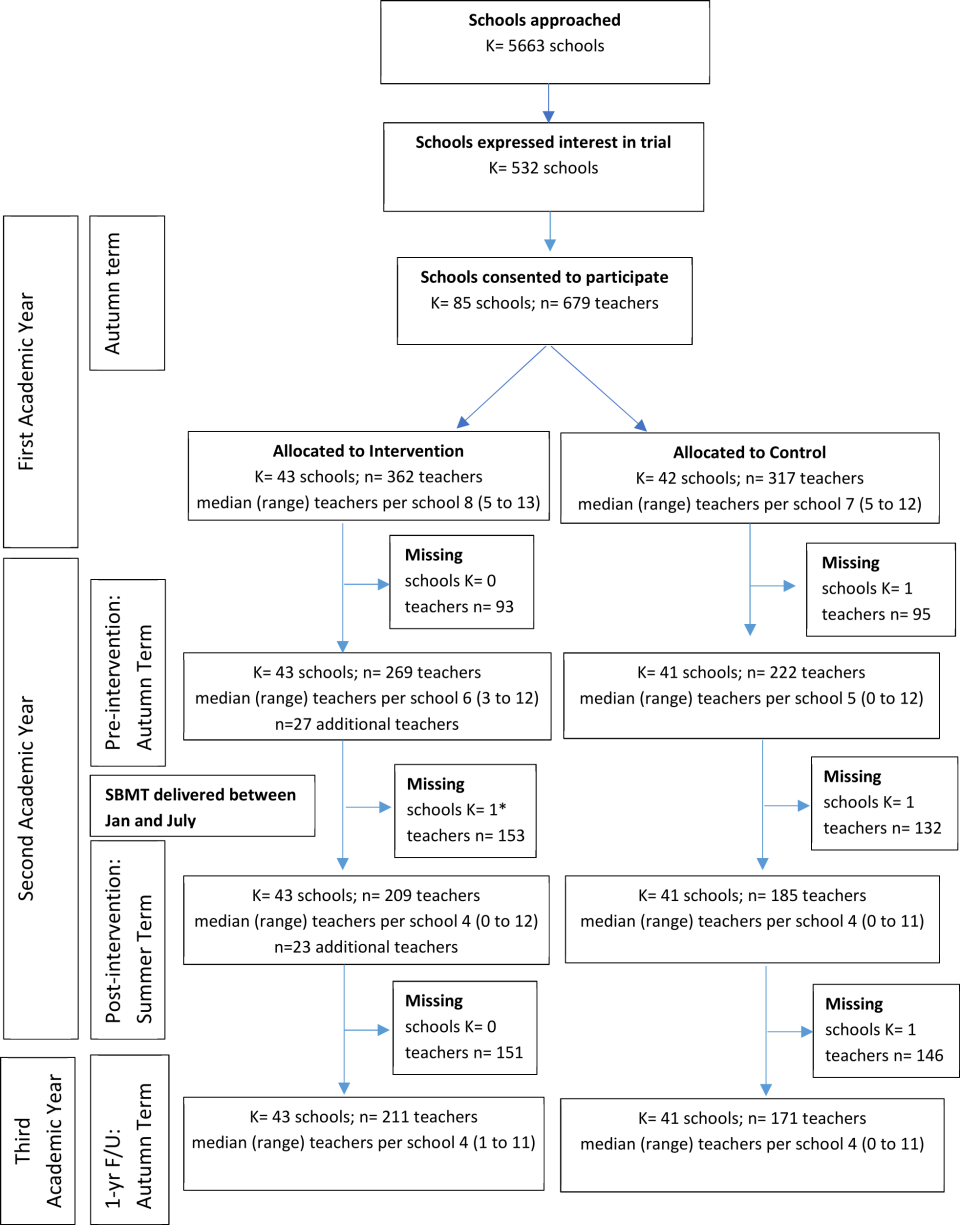
Consolidated Standards of Reporting Trials from diagram for trial schools and teachers. ‘Missing’ is the number of schools (K)/teachers (n) that did not provide data on any of the outcomes at the subsequent timepoint. Teachers could be temporarily lost to follow-up if not in school for a given timepoint. Six hundred seventy-nine teachers in total were recruited, but seven were from a school which dropped out. So only 672 teachers were available for analysis. *None of the teachers at one school provided any follow-up data at the postintervention timepoint. However, teachers from this school provided data at the 1-year follow-up. First Academic Year-Autumn term: prerandomisation (baseline); Preintervention-Autumn Term: postpersonal mindfulness (MBCT-L) and school-based mindfulness training (SBMT) teacher training; Post intervention-Summer Term: after delivery of the SBMT to students; 1-yrF/U-Autumn Term: 1-year follow-up measure.

**Figure 2:**
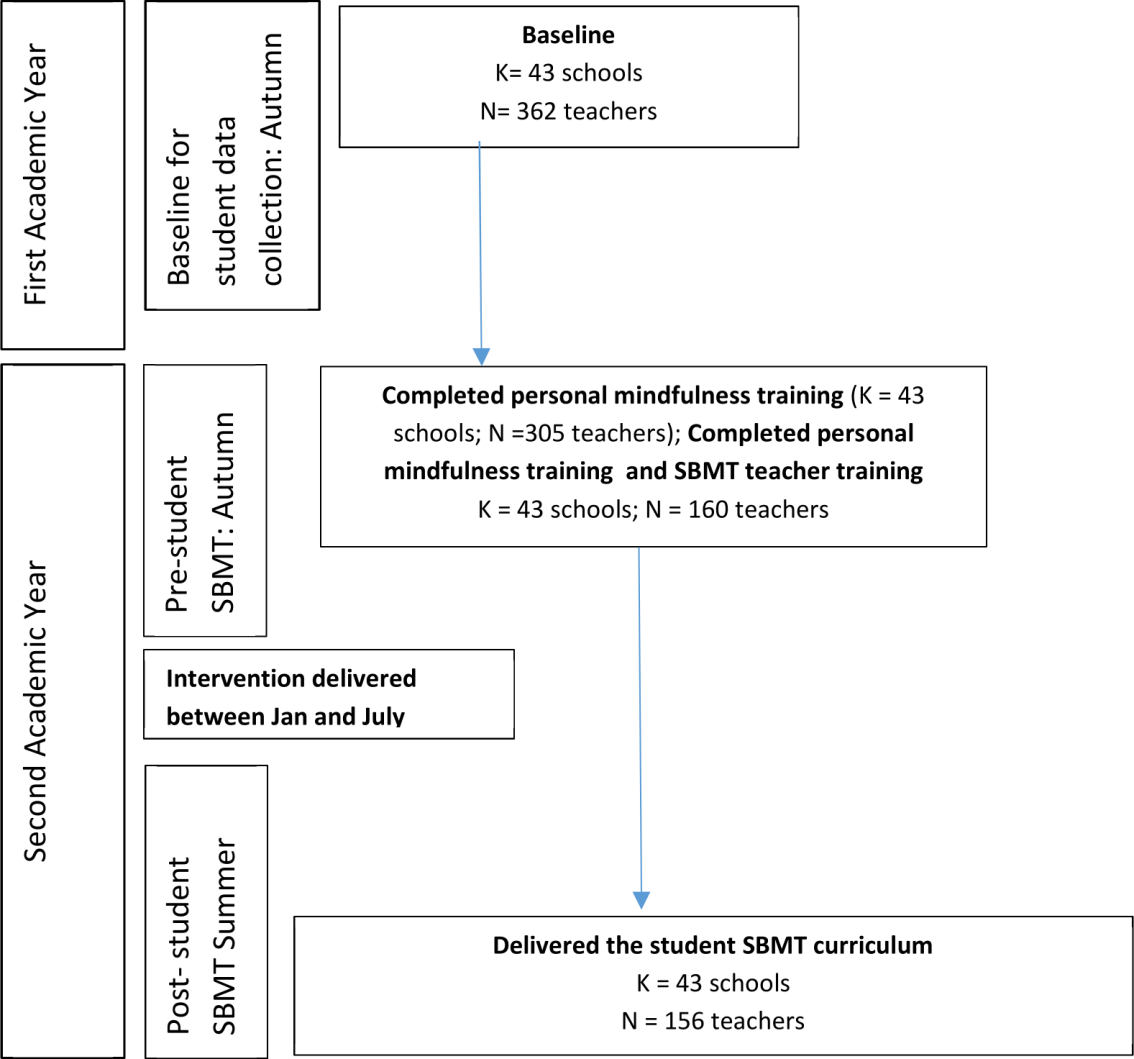
Teacher selection through the school-based mindfulness training. This represents teacher flow through the school-based mindfulness training (SBMT) and implementation, and is explained in the Methods and Results sections. Teacher attrition is shown in figure 1. Baseline for student data collection-autumn: prerandomisation (baseline); prestudent SBMT-autumn: postpersonal mindfulness (MBCT-L) and SBMT teacher training; post-student SBMT-summer: after delivery of the SBMT to students.

TAU schools offered SEL in a wide variety of ways, and the participating study teachers were not necessarily involved in this delivery. Teacher recruitment and attrition across both trial arms are described in figure 1. Teacher selection within the SBMT arm is described in figure 2. Due to the nature of SBMT, teachers were not blind to intervention allocation. The trial data file was cleaned, locked and signed-off by the Trial Steering and Data Monitoring Committees before the statistician was unblinded.

### Setting

The study setting was UK secondary schools offering SEL in line with good practice.

### Randomisation

Following collection of baseline data, schools were randomised (1:1) to trial arms based on computer generated random numbers. An independent statistician generated the allocation sequence, and the trial manager enrolled the clusters and assigned them to the trial arms. Allocation of schools was balanced on: school size (large (≥1000 children), small (<1000 children)); school quality (‘outstanding/good’, ‘requires improvement’); and deprivation (below/above the median percentage across all UK schools of children eligible for free school meals; 29.4%). For cohort 2, allocation was also balanced on type of school (boys, girls, mixed) and region (England, Scotland, Wales, Northern Ireland). Constrained randomisation^21^ was used, where the allocation sequence was randomly selected out of those with a high level of balance on the above factors between arms. Allocation concealment was achieved as all schools (and teachers) were recruited before randomisation and allocated en bloc for each cohort.^17 18^

### Interventions

#### SBMT programme

While the SBMT programme’s primary aim was to teach mindfulness skills that support young people’s resilience, its wider aims included enhancing teachers’ mental health and school climate. Informed by theory and implementation science,^16^ SBMT was designed to be integrated into the school curriculum. The process started with engaging the school leadership team, and then identifying a potential pool of teachers from within the school who could be trained and timetabled to deliver it to the students.

The full SBMT teacher training involved first participating in an 8-week personal mindfulness-based cognitive therapy for life (MBCT-L) programme. MBCT-L is an adaptation of the original MBCT programme which was developed for people with a history of recurrent depression.^22^ It is an 8-week, group-based (up to 15 participants), psychoeducational programme that integrates cognitive–behavioural strategies with MT. Each weekly session was 2 hours long and participants were asked to complete 40 min per day of mindfulness practice. The programme includes strategies and practices to stabilise attention, regulate emotions and behaviours, enhance self-care and transfer this learning into personal/professional domains. Sessions included guided mindfulness practices, weekly homework and teaching/discussion.

From the pool of teachers undergoing personal MT (MBCT-L), schools selected the sample of teachers to go forward with the SBMT. Senior leadership teams in schools based their selection on whether teachers would be willing and available to attend further training and could be timetabled to teach the SBMT to participating study classes. Selected teachers then attended a 4-day training workshop to learn how to deliver SBMT to students. Following this 4-day training, participating teachers taught the full SBMT curriculum to a group of non-participating pupils (non-study pupils) within the same school before delivering it to participating pupils (study pupils), with support from an experienced mentor, before going on to teach the study students.

Within participating schools, as many teachers as possible were invited to attend the personal MT (MBCT-L), to give schools the best opportunity to timetable the required number of teachers to teach the SBMT curriculum to study classes. Further embedding SBMT into the school included opening the training up to staff beyond the nominated teachers, helping schools integrate mindfulness into their school improvement plan and using mindfulness skills throughout the school curriculum.

#### Teaching as usual

The trial aim was to establish if SBMT is a potential alternative to current good SEL practice in secondary schools. Therefore, no additional teacher training or SEL was provided in the TAU arm. There is a large variety of SEL programmes^23^ and delivering SEL is rarely mandatory. In the UK, it is usually taught as part of ‘Personal, Social, Health and Economic Education’ intended to prepare students with the knowledge, skills and attributes they need to manage their lives. While SEL provision was not uniform in the TAU arm, TAU schools agreed not to provide other curricula that include MT until study completion. SEL provision was assessed using a bespoke tool based in part on existing measures (see online supplemental A and B for more details).

10.1136/ebmental-2022-300424.supp1 Supplementary data

### Outcome measures

Outcomes were measured at the teacher level prerandomisation (baseline−T0), post personal MT (MBCT-L) and SBMT teacher training (or equivalent time in the TAU arm−T1), after delivery of the SBMT to students (T2), and 1-year follow-up after post personal MT (MBCT-L) and SBMT teacher training (2 years after baseline−T3).

Teacher mental health was assessed with the following measures: teachers’ burnout (Maslach Burnout Inventory-Educator Survey, MBI-ES, including exhaustion, depersonalisation and (lack of) personal accomplishment); self-efficacy (Teacher’s Self-Efficacy Scale, including student engagement, instructional practice and classroom management); mindfulness (Five Facet Mindfulness Questionnaire-Short Form); mindfulness in teaching (Mindfulness in Teaching Scale, including intrapersonal and interpersonal mindfulness); stress (Perceived Stress Scale, PSS); depression (Patient Health Questionnaire); and anxiety (Generalised Anxiety Disorder).

School climate was assessed with the School Climate and Connectedness Survey, using three scales most relevant to SBMT: ‘School Leadership and Involvement,’ ‘Staff Attitudes,’ and ‘Respectful Climate’. All participating study teachers (only those that participated in the trial) rated school climate, and the average teacher rating for each school was used.

We assessed the participating teachers’ engagement with the SBMT in two ways. First their personal MT (MBCT-L) was assessed through two self-report items asking about the frequency of formal (structured) and informal (flexible) mindfulness practice. Second, the extent to which participating teachers completed the full training/implementation route of the SBMT was recorded. Further details on the measures are in online supplemental A.

### Statistical analysis

The analysis followed a prespecified plan. The sample size for the trial was justified on the basis of student outcomes,^17^ so no formal power calculations were undertaken regarding teacher outcomes.

Characteristics were summarised using means (SDs) for continuous variables and numbers (percentages) for categorical variables. Outcomes were compared between the trial arms using the intention-to-treat principle. Missing outcome data (assumed to be missing at random) were imputed using the multivariate linear mixed effects (‘multilevel’) model. The imputation model included the outcomes at each time point, trial arm, the stratification factors and other characteristics prespecified for covariate adjustment, the extent to which formal/informal mindfulness was practised, and the completion of the full training/implementation route of the SBMT, generating 20 imputed datasets.

Outcomes at each time point were compared using mixed effects linear regressions, allowing for correlation between observations from the same school (cluster). The main comparisons were adjusted for the factors used to balance the randomisation, study cohort, and the baseline score for the outcome. Means (SDs) were reported for each trial arm along with the crude mean difference between arms, the adjusted mean difference, the 95% CI and p value for the adjusted mean difference. The pooled intracluster correlation coefficient across the trial arms was reported for the outcomes from the crude (unadjusted) analysis. Results from the complete case analyses are also reported.

Additional *post hoc* sensitivity analyses utilising Complier Average Causal Effect (CACE)/instrumental variable methods^24^ were also undertaken to explore the causal effects of: (1) teacher engagement with the SBMT intervention and (2) regular formal/informal mindfulness practice. For the former, two definitions of compliance (engagement) were used: (1) attendance at a minimum of four personal MT (MBCT-L) sessions; and (2) attendance at a minimum of four personal MT (MBCT-L) sessions *and* the 4-day training course *and* delivery of at least one mindfulness session to students. For the assessment of the effects of both formal and informal mindfulness practice, three definitions of compliance (self-reported mindfulness practice) were used: (1) at least occasionally; (2) at least several times per week, and (3) daily. A two-stage least squares instrumental variable approach with cluster (school) robust standard errors was used to estimate these causal effects.^24^ Teacher gender, number of years teaching and preintervention stress (measured using PSS) were included at the first stage regression (to predict compliance), and adjustments for cohort, the factors used to balance the randomisation, and the preintervention value of the outcome were included at the second stage regression (to predict the outcome). Allocated group was used as the instrumental variable for each of the compliance definitions. All mean differences are presented with 95% CIs and p values. Unadjusted mean differences, without adjustment at either stage of the two-stage least squares model, are also reported.

Data were imputed and analysed using R (V.3.6.1),^25^ and Stata V.16.1, respectively.

## Findings

We recruited 85 schools; 43 were randomised to SBMT and 42 to TAU. Six hundred seventy-nine teachers were recruited: 362 in the SBMT arm and 317 in the TAU arm. One school, including seven teachers, withdrew soon after randomisation because of the time commitment and was therefore excluded. As a result, 672 teachers (362 SBMT, 310 TAU) and 84 schools (43 SBMT, 41 TAU) were included in the analyses. Schools and teachers in both trial arms are described in table 1. Participating schools were broadly representative of UK schools (online supplemental B) with respect to the population served on key variables such as deprivation (ie, percentage of children eligible for free school meals), and the type of school (ie, selective/non-selective, urban/rural, large/small, mixed/single gender, state maintained/independent).^19 26^

**Table 1:** Preintervention school and teacher characteristics by trial arm status and overall

	SBMT arm	TAU arm	Total
School characteristics	**K=43**	**K=42**	**K=85**
Region			
England, n (%)	38 (88)	37 (88)	75 (88)
Scotland, n (%)	1 (2)	2 (5)	3 (4)
Wales, n (%)	2 (5)	1 (2)	3 (4)
Northern Ireland, n (%)	2 (5)	2 (5)	4 (5)
School size—at least 1000 students, n (%)	20 (47)	23 (55)	43 (51)
Type of school			
Mixed, n (%)	36 (84)	38 (90)	74 (87)
Girls, n (%)	7 (16)	4 (10)	11 (13)
School quality rating			
Requires improvement, n (%)	6 (14)	5 (12)	11 (13)
Outstanding/good, n (%)	37 (86)	37 (88)	74 (87)
Deprivation			
Above median percentage eligible for free school meals, n (%)	15 (35)	15 (36)	30 (35)
Below median percentage eligible for free school meals, n (%)	28 (65)	27 (64)	55 (65)
Provision of Social Emotional Learning (SEL), mean (SD)	12 (2.5)	12 (2.6)	12 (2.6)
**Teacher characteristics**	**N=362**	**N=317**	**N=679**
Age, mean (SD)	40.2 (8.9)	39.1 (9.2)	39.7 (9.0)
Gender			
Female, n (%)	283 (78.4)	223 (70.4)	506 (74.6)
Male, n (%)	77 (21.3)	93 (29.3)	170 (25.1)
Other, n (%)	1 (0.3)	1 (0.3)	2 (0.3)
Ethnicity			
Arab/Arab British, n (%)	0 (0.0)	2 (0.6)	2 (0.3)
Asian/British Asian, n (%)	7 (1.9)	13 (4.1)	20 (3.0)
Black/African/Caribbean, n (%)	2 (0.6)	3 (1.0)	5 (0.7)
Mixed/Multiple Ethnic Group, n (%)	9 (2.5)	4 (1.3)	13 (1.9)
White, n (%)	341 (94.5)	292 (92.1)	633 (93.4)
Other Ethnic Group, n (%)	2 (0.6)	3 (1.0)	5 (0.7)
Qualified teacher status, n (%)	353 (97.8)	309 (97.5)	662 (97.6)
Years teaching experience, mean (SD)	12.9 (8.5)	12.2 (8.6)	12.6 (8.6)
Experience of mindfulness			
Currently practising mindfulness, n (%)	39 (10.8)	21 (6.6)	60 (8.9)
Some understanding of mindfulness, n (%)	291 (80.6)	268 (84.5)	559 (82.5)
Not aware of mindfulness, n (%)	31 (8.6)	28 (8.8)	59 (8.7)
School Climate and Connectedness Survey (SCCS)			
School leadership and involvement, mean (SD)	3.9 (0.6)	3.9 (0.6)	3.9 (0.6)
Staff attitudes, mean (SD)	4.1 (0.6)	4.2 (0.6)	4.2 (0.6)
Respectful climate, mean (SD)	3.8 (0.6)	3.8 (0.6)	3.8 (0.6)
Maslach Burnout Inventory (MBI Educators Survey)			
Emotional exhaustion, mean (SD)	2.4 (1.2)	2.3 (1.2)	2.4 (1.2)
Depersonalisation, mean (SD)	0.7 (0.7)	0.7 (0.8)	0.7 (0.8)
Personal accomplishment, mean (SD)	1.0 (0.7)	1.1 (0.8)	1.0 (0.7)
Mindfulness in Teaching Scale (MTS)			
Interpersonal, mean (SD)	20.6 (2.5)	20.6 (2.6)	20.6 (2.6)
Intrapersonal, mean (SD)	33.4 (5.2)	34.0 (5.6)	33.7 (5.4)
Perceived Stress Scale (PSS), mean (SD)	16.4 (7.0)	16.5 (7.2)	16.4 (7.1)
Patient Health Questionnaire (PHQ-9), mean (SD)	5.2 (4.3)	5.2 (4.6)	5.2 (4.4)
Generalised Anxiety Disorder—7 (GAD-7), mean (SD)	4.3 (4.0)	4.7 (4.5)	4.5 (4.2)
Five Facet Mindfulness Questionnaire (FFMQ-SF), mean (SD)	83.6 (12.6)	84.7 (13.4)	84.1 (13.0)
Teachers' Sense of Efficacy Scale (TSES)			
Student engagement, mean (SD)	6.9 (1.1)	6.8 (1.1)	6.9 (1.1)
Instructional practice, mean (SD)	7.3 (1.0)	7.4 (1.0)	7.3 (1.0)
Classroom management, mean (SD)	7.6 (1.0)	7.5 (1.0)	7.6 (1.0)

Data on preintervention school characteristics are provided for all schools in the SBMT arm. Of the 42 schools, 41 in the TAU provided data on SEL provision. Data on all other preintervention school characteristics are provided for all schools in the TAU arm. No all boys schools were recruited. Sample size ranges from 360 to 362 teachers in the SBMT arm and 316 to 317 teachers in the TAU arm. In the SBMT arm, all 362 teachers provided data on the SCCS respectful climate subscale and MBI subscales. Three hundred sixty-one teachers provided data on age, gender, ethnicity, qualified teacher status, years teaching experience, experience of mindfulness, SCCS school leadership and involvement and staff attitudes subscales, PSS, PHQ-9, GAD-7, FFMQ-SF and TSES subscales. Three hundred sixty teachers provided data on MTS subscales. In the TAU arm, all 317 teachers provided data on gender, ethnicity, qualified teacher status, years teaching experience, experience of mindfulness and SCCS subscales. Three hundred sixteen teachers provided data on age, MBI subscales, MTS subscales, PSS, PHQ-9, GAD-7, FFMQ-SF and TSES subscales.

The number of schools and teachers that were lost to follow-up at each time point are reported in figure 1 (online supplemental tables S1–S3). Within the SBMT arm, there were teachers who did not progress through the full SBMT training/implementation, due to timetabling issues. Teachers across both arms were lost to follow-up through being off school (eg, maternity or sick leave), or teachers being otherwise unreachable. In general, teachers lost to follow-up were younger, had fewer years of teaching experience, were less likely to be White, and were more likely to be qualified teachers. Teachers lost to follow-up, compared with those who remained, were less likely to be female in the SBMT arm, but more likely to be female in the TAU arm (online supplemental tables S1–S3).

### Teacher outcomes

Following their personal MT (MBCT-L) and teacher training, teachers in the SBMT arm reported greater student engagement (self-efficacy) than teachers in the TAU arm, but differences for the other two subscales were not significant (online supplemental table S4). After delivering the curriculum to students, teachers in the SBMT arm reported less burnout (emotional exhaustion and lack of personal accomplishment), but differences for depersonalisation were only significant when a minimum of attendance was present (online supplemental table S5). There was little evidence of a difference on other measures of mental health, perceived stress, self-efficacy, or mindfulness between teachers in the SBMT and TAU arms at either of these time points (online supplemental tables S4 and S5). At 1-year follow-up, there was only evidence of differences between teachers in the SBMT on mindfulness intrapersonal compared with teachers in the TAU arm (table 2).

**Table 2:** Main comparisons of teacher outcomes at 1-year follow-up

Outcome	SBMT arm (I)		TAU arm (C)		Unadjusted mean Difference (I-C)	Adjusted mean difference (I-C)			ICC*
	N	Mean (SD)	N	Mean (SD)	Estimate	Estimate	95% CI	P value	
Mental health – (MBI)									
Exhaustion									
*ITT*	210	2.4 (1.2)	171	2.5 (1.2)	−0.1	−0.1	−0.3 to 0.1	0.247	0.080
*CACE (i)†*					−0.1	−0.2	−0.4 to 0.1	0.221	–
*CACE (ii)‡*					−0.3	−0.4	−0.9 to 0.1	0.154	–
Depersonalisation									
*ITT*	210	0.7 (0.7)	171	0.9 (0.9)	−0.1	−0.1	−0.2 to 0.1	0.362	0.139
*CACE (i)†*					−0.1	−0.1	−0.3 to 0.1	0.274	–
*CACE (ii)‡*					−0.3	−0.3	−0.7 to 0.1	0.147	–
Personal accomplishment									
*ITT*	210	1.0 (0.7)	171	1.1 (0.8)	−0.1	−0.1	−0.2 to 0.1	0.258	0.025
*CACE (i)†*					−0.1	−0.1	−0.3 to 0.1	0.208	–
*CACE (ii)‡*					−0.3	−0.3	−0.6 to 0.1	0.142	–
Self-efficacy Questionnaire (TSES)									
Student Engagement Subscale									
*ITT*	204	7.0 (1.1)	162	6.8 (1.1)	0.2	0.1	−0.1 to 0.3	0.269	0.133
*CACE (i)†*					0.2	0.1	−0.1 to 0.4	0.262	–
*CACE (ii)‡*					0.4	0.4	−0.2 to 0.9	0.188	–
Instructional Practice Subscale									
*ITT*	204	7.5 (0.8)	162	7.5 (1.0)	0.001	0.03	−0.1 to 0.2	0.704	0.087
*CACE (i)†*					−0.002	0.04	−0.2 to 0.2	0.737	–
*CACE (ii)‡*					−0.004	0.1	−0.3 to 0.6	0.577	–
Classroom Management Subscale									
*ITT*	204	7.8 (0.8)	162	7.6 (0.9)	0.2	0.1	−0.04 to 0.3	0.132	0.099
*CACE (i)†*					0.2	0.2	−0.05 to 0.4	0.116	–
*CACE (ii)‡*					0.5	0.5	−0.02 to 1.0	0.060	–
Mindfulness (FFMQ-SF)									
*ITT*	205	85.4 (12.8)	162	86.1 (13.9)	−0.8	−0.2	−2.4 to 2.0	0.854	0.028
*CACE (i)†*					−1.2	−0.1	−2.9 to 2.6	0.935	–
*CACE (ii)‡*					−2.6	1.0	−4.5 to 6.6	0.711	–
Mindfulness (MTS)—Interpersonal									
*ITT*	208	20.8 (2.7)	168	20.9 (2.4)	−0.2	−0.2	−0.7 to 0.2	0.333	0.028
*CACE (i)†*					−0.3	−0.3	−0.9 to 0.3	0.318	–
*CACE (ii)‡*					−0.7	−0.4	−1.5 to 0.8	0.522	–
Mindfulness (MTS)—Intrapersonal									
*ITT*	208	32.1 (5.0)	168	33.4 (5.6)	−1.2	−0.9	−1.7 to −0.1	0.020	0.060
*CACE (i)†*					−1.7	−1.2	−2.2 to −0.2	0.024	–
*CACE (ii)‡*					−3.7	−2.0	−4.1 to 0.03	0.053	–
Stress (PSS)									
*ITT*	207	15.3 (6.9)	165	15.9 (7.4)	−0.3	0.01	−1.1 to 1.2	0.982	0.075
*CACE (i)†*					−0.3	−0.1	−1.7 to 1.4	0.890	–
*CACE (ii)‡*					−0.6	−0.6	−3.8 to 2.5	0.686	–
Depression (PHQ-9)									
*ITT*	206	4.7 (4.1)	164	5.0 (4.2)	−0.3	−0.1	−1.0 to 0.7	0.731	0.052
*CACE (i)†*					−0.4	−0.2	−1.3 to 0.9	0.712	–
*CACE (ii)‡*					−0.9	−0.9	−3.1 to 1.3	0.438	–
Anxiety (GAD7)									
*ITT*	206	4.2 (3.9)	163	4.3 (4.1)	−0.1	0.2	−0.7 to 1.0	0.696	0.045
*CACE (i)†*					−0.1	0.2	−0.9 to 1.3	0.742	–
*CACE (ii)‡*					−0.3	0.2	−2.1 to 2.5	0.867	–
*School ecology/climate (SCCS)*									
School leadership and involvement									
*ITT*	211	3.9 (0.7)	171	3.8 (0.7)	0.1	0.1	−0.04 to 0.3	0.144	0.248
*CACE (i)†*					0.2	0.2	−0.03 to 0.4	0.100	–
*CACE (ii)‡*					0.3	0.4	−0.04 to 0.8	0.079	–
Staff attitudes									
*ITT*	211	4.1 (0.6)	171	4.1 (0.5)	0.03	0.1	−0.1 to 0.2	0.253	0.280
*CACE (i)†*					0.03	0.1	−0.1 to 0.3	0.207	–
*CACE (ii)‡*					0.1	0.2	−0.1 to 0.6	0.165	–
Respectful Climate									
*ITT*	211	3.9 (0.5)	171	3.7 (0.6)	0.1	0.2	0.02 to 0.3	0.020	0.274
*CACE (i)†*					0.2	0.2	0.04 to 0.4	0.015	–
*CACE (ii)‡*					0.4	0.4	0.1 to 0.8	0.012	–

*Intracluster (intraschool) correlation coefficients (ICCs) from crude (unadjusted) analyses.

†A participant in the SBMT arm is deemed a complier if they have attended at least four of the eight personal mindfulness (MBCT-L) sessions.

‡A participant in the SBMT arm is deemed a complier if they have attended at least four of the eight personal mindfulness (MBCT-L) sessions and the 4-day training course, and have delivered at least one mindfulness session.

FFMQ-SF, Five Facet Mindfulness Questionnaire-Short Form; GAD7, Generalised Anxiety Disorder; MBI, Maslach Burnout Inventory; MTS, Mindfulness in Teaching Scale; PHQ-9, Patient Health Questionnaire; PSS, Perceived Stress Scale; SCCS, School Climate and Connectedness Survey; TSES, Teacher’s Self-Efficacy Scale.

### School outcomes

Following their personal MT (MBCT-L), teachers in the SBMT arm reported better school leadership and involvement than teachers in the TAU arm (online supplemental table S4). After delivering the SBMT to students, teachers in the SBMT arm continued to report better school climate in terms of leadership and involvement, but also in respectful climate (online supplemental table S5). At 1-year follow-up, teachers in the SBMT arm continued to report a more respectful climate than teachers in the TAU arm, but there were no differences in other dimensions of school climate (table 2).

### Teachers’ engagement with the SBMT and outcomes

In the SBMT arm, 84% (305/362) of teachers attended personal MT (MBCT-L); while 44% (160/362) of teachers attended personal MT (MBCT-L) and SBMT teacher training. Regarding *formal mindfulness practice* at 1-year follow-up, 69% (140/204) practised mindfulness at least occasionally, 24% (48/204) at least several times a week and 4% (8/204) practised daily. For *informal mindfulness practice* at 1-year follow-up, 82% (167/204) practised mindfulness at least occasionally, 48% (97/204) at least several times a week and 18% (36/204) practised daily.

The results of the CACE/instrumental variable analyses represent estimation of the intervention effect among the subpopulation of compliers in the intervention arm, compared with those in the control arm who *would* have complied with the intervention, had they been offered it. The CACE analyses and the instrumental variable analyses indicated little evidence of a relationship between personal mindfulness (attendance at the 8-week MBCT-L course) (table 2, online supplemental tables S4 and S5) or formal or informal mindfulness (online supplemental tables S6–S11) and the outcomes. As with the ITT analysis, the only exceptions were intrapersonal mindfulness and respectful climate at 1 year, and burnout and school climate at post intervention.

Main comparisons of teacher outcomes using complete case analysis are provided in online supplemental tables S12–14.

### Adverse events

Two serious adverse events were recorded. The independent Data Monitoring and Ethics Committee concluded that neither was attributable to the SBMT.

## Discussion

This study extends our earlier report of a universal SBMT compared with standard SEL on student mental health and well-being by focussing on teachers. SBMT, compared with usual provision, reduced teacher burnout with small effects. This finding is noteworthy as burnout is a significant hazard in the teaching profession.^27^ However, effects were not extended to other teacher outcomes and were not sustained at 1-year follow-up.

SBMT compared with TAU was associated with enhancements in school climate, at least as perceived by the teachers themselves. These differences in school climate in terms of respectful climate were maintained at 1-year follow-up with small-to-moderate effects. This is important because a positive school environment could create a cascade of effects, for example, improving teacher–student relationships and enabling more positive staff relationships.^11 12^ In contrast, a poor school climate may create a ‘burnout cascade’: job-related exhaustion, depersonalisation and demoralisation.^13^

This is a report of secondary outcomes of the MYRIAD trial and as such the findings are exploratory and subject to several limitations (eg, the multiple analyses may lead to statistical significance through chance alone). The school climate measure was a rating by study teachers, and not all teachers in the school; they may be subject to selection/demand effects. It would be interesting to crossvalidate this with wider perspectives, for example, teacher absenteeism. Finally, participating teachers reported above average mental health and this likely facilitated their SBMT entry. Engagement with mindfulness practice typically is associated with greater effects, but also people with better well-being may be less motivated to practice. It is important in interpreting and generalising the findings to consider which teachers are best placed to teach SEL.

Some SEL programmes are primarily student-oriented and hence effects on teacher mental health would likely be indirect. Others like this universal SBMT are intended to include school and teacher level effects, both directly through personal MT and indirectly through changes in school climate. Theoretical models that articulate how universal SEL programmes might create transformative and lasting change have been articulated,^2 13 16^ and now need systematic testing if we are to create schools that not only advance students’ academic learning but also support the well-being of the whole school community.

## Clinical implications

The analyses reported in this paper indicate that SBMT might have promise in supporting changes in teacher burnout and school climate. However, innovation is required if broader effects are to manifest and be preserved.^16 28^

## Data Availability

Data are available upon reasonable request. The baseline data and codebook from the MYRIAD trial are available from Prof. Kuyken (willem.kuyken@psych.ox.ac.uk) upon request (release of data is subject to an approved proposal and a signed data access agreement).

## References

[1] Bonell C , Humphrey N , Fletcher A , et al . Why schools should promote students' health and wellbeing. BMJ 2014;348:g3078. 10.1136/bmj.g3078 25134103

[2] Domitrovich CE , Bradshaw CP , Greenberg MT , et al . Integrated models of school-based prevention: logic and theory. Psychol Sch 2010;47:71–88. 10.1002/pits.20452 27182089 PMC4865396

[3] Zins J , Elias M , Greenberg M . Facilitating success in school and in life through social and emotional learning. Perspectives in Education 2003;21:55–67.

[4] Durlak JA , Weissberg RP , Dymnicki AB , et al . The impact of enhancing students' social and emotional learning: a meta-analysis of school-based universal interventions. Child Dev 2011;82:405–32. 10.1111/j.1467-8624.2010.01564.x 21291449

[5] Wang M-T , Degol JL . School climate: a review of the construct, measurement, and impact on student outcomes. Educ Psychol Rev 2016;28:315–52. 10.1007/s10648-015-9319-1

[6] Thapa A , Cohen J , Guffey S , et al . A review of school climate research. Rev Educ Res 2013;83:357–85. 10.3102/0034654313483907

[7] Cohen J , Mccabe EM , Michelli NM , et al . School climate: research, policy, practice, and teacher education. Teach Coll Rec 2009;111:180–213. 10.1177/016146810911100108

[8] Kidger J , Brockman R , Tilling K , et al . Teachers' wellbeing and depressive symptoms, and associated risk factors: a large cross sectional study in English secondary schools. J Affect Disord 2016;192:76–82. 10.1016/j.jad.2015.11.054 26707351

[9] Hakanen JJ , Bakker AB , Schaufeli WB . Burnout and work engagement among teachers. J Sch Psychol 2006;43:495–513. 10.1016/j.jsp.2005.11.001

[10] Greenberg MT , Domitrovich CE , Graczyk PA . The study of implementation in school-based preventive interventions: theory, research, and practice. Rockville, MD: Center for Mental Health Services, Substance Abuse and Mental Health Services Administration, 2005.

[11] Kidger J , Donovan JL , Biddle L , et al . Supporting adolescent emotional health in schools: a mixed methods study of student and staff views in England. BMC Public Health 2009;9:403. 10.1186/1471-2458-9-403 19878601 PMC2777165

[12] Singh K , Billingsley BS . Professional Support and Its Effects on Teachers’ Commitment. J Educ Res 1998;91:229–39. 10.1080/00220679809597548

[13] Jennings PA , Greenberg MT . The prosocial classroom: teacher social and emotional competence in relation to student and classroom outcomes. Rev Educ Res 2009;79:491–525. 10.3102/0034654308325693

[14] Dunning DL , Griffiths K , Kuyken W , et al . Research Review: The effects of mindfulness-based interventions on cognition and mental health in children and adolescents - a meta-analysis of randomized controlled trials. J Child Psychol Psychiatry 2019;60:244–58. 10.1111/jcpp.12980 30345511 PMC6546608

[15] Lomas T , Medina JC , Ivtzan I , et al . The impact of mindfulness on the wellbeing and performance of educators: a systematic review of the empirical literature. Teach Teach Educ 2017;61:132–41. 10.1016/j.tate.2016.10.008

[16] Tudor K , Maloney S , Raja A , et al . Universal mindfulness training in schools for adolescents: a scoping review and conceptual model of Moderators, mediators, and implementation factors. Prev Sci 2022. doi:10.1007/s11121-022-01361-9. [Epub ahead of print: 10 Mar 2022]. PMC9343282 35267177

[17] Kuyken W , Nuthall E , Byford S , et al . The effectiveness and cost-effectiveness of a mindfulness training programme in schools compared with normal school provision (Myriad): study protocol for a randomised controlled trial. Trials 2017;18:194. 10.1186/s13063-017-1917-4 28446223 PMC5406917

[18] Montero-Marin J , Nuthall E , Byford S , et al . Update to the effectiveness and cost-effectiveness of a mindfulness training programme in schools compared with normal school provision (Myriad): study protocol for a randomised controlled trial. Trials 2021;22:254. 10.1186/s13063-021-05213-9 33827652 PMC8024679

[19] Kuyken W , Ball S , Crane C . Effectiveness and cost-effectiveness of a universal school-based mindfulness training programme compared with normal school provision in preventing mental health problems and promoting well-being: results of the Myriad cluster randomised controlled trial. manuscript under review, 2021.

[20] Campbell MK , Elbourne DR , Altman DG , et al . Consort statement: extension to cluster randomised trials. BMJ 2004;328:702–8. 10.1136/bmj.328.7441.702 15031246 PMC381234

[21] Moulton LH . Covariate-based constrained randomization of group-randomized trials. Clin Trials 2004;1:297–305. 10.1191/1740774504cn024oa 16279255

[22] Segal ZV , Williams JMG , Teasdale JD . Mindfulness-based cognitive therapy for depression. Second edition. New York: Guilford Press, 2013.

[23] Durlak JA , Domitrovich CE , Weissberg RP , et al . Handbook of social and emotional learning: research and practice. New York: The Guilford Press, 2015.

[24] Dunn G , Bentall R . Modelling treatment-effect heterogeneity in randomized controlled trials of complex interventions (psychological treatments). Stat Med 2007;26:4719–45. 10.1002/sim.2891 17476649

[25] Grund S , Ludtke O , Robitzsch A . Multiple imputation of multilevel missing data: an introduction to the R package pan. Sage Open 2016;6:4.

[26] Ford T , Degli Esposti M , Crane C , et al . The role of schools in early adolescents' mental health: findings from the Myriad study. J Am Acad Child Adolesc Psychiatry 2021;60:1467–78. 10.1016/j.jaac.2021.02.016 33677037 PMC8669152

[27] Montero-Marin J , Taylor L , Crane C , et al . Teachers "Finding Peace in a Frantic World": An Experimental Study of Self-Taught and Instructor-Led Mindfulness Program Formats on Acceptability, Effectiveness, and Mechanisms. J Educ Psychol 2021;113:1689–708. 10.1037/edu0000542 34912129 PMC8647626

[28] Wilde S , Sonley A , Crane C , et al . Mindfulness training in UK secondary schools: a multiple case study approach to identification of Cornerstones of implementation. Mindfulness 2019;10:376–89. 10.1007/s12671-018-0982-4 31186817 PMC6558285

