# Supplementary material for: Effectiveness of universal school-based mindfulness training compared with normal school provision on teacher mental health and school climate: results of the MYRIAD cluster randomised controlled trial
Source: Evid Based Ment Health. 2022 Jul 7;25(3):125–34. doi: 10.1136/ebmental-2022-300424 (PMC9340006; doi:10.1136/ebmental-2022-300424)
Supplement: Supplementary data [file ebmental-2022-300424supp001.pdf]

Effectiveness of a universal school-based mindfulness training compared with normal school provision on teacher well-being and school climate: results of the MYRIAD cluster randomised controlled trial

Supplements

Supplement A. Teaching as usual: social-emotional learning

Supplement B. School and Teacher measures

Supplement C. Representativeness of study schools

Supplementary Table S1: Selected teacher baseline characteristics at post-personal MT follow-up for remaining teachers and teachers lost to follow up

Supplementary Table S2: Selected teacher baseline characteristics at post SBMT teacher training for remaining teachers and teachers lost to follow up

Supplementary Table S3: Selected teachers baseline characteristics at 1-year follow-up status for remaining teachers and teachers lost to follow-up

Supplementary Table S4: Main comparisons of teacher outcomes following personal MT, but before delivery of the student MT in the SBMT arm, and at comparable timepoint in TAU arm

Supplementary Table S5: Main comparisons of teacher outcomes post teacher training and delivery of the pupil MT

Supplementary Table S6: Instrumental Variable Analysis of the teacher outcomes at 1-year follow-up, with allocated group an instrument for formal mindfulness practice

Supplementary Table S7: Instrumental Variable Analysis of the teacher outcomes at 1-year follow-up, with allocated group an instrument for informal mindfulness practice

Supplementary Table S8: Instrumental Variable Analysis of the teacher outcomes following SBMT teacher training but before delivery of the pupil MT, with allocated group an instrument for formal mindfulness practice

Supplement Table S9: Instrumental Variable Analysis of the teacher outcomes following teacher training, but before the pupil MT, with allocated group an instrument for informal mindfulness practice

Supplement Table S10: Instrumental Variable Analysis of the teacher outcomes following the teacher training and delivery of the pupil MT curriculum, with allocated group an instrument for formal mindfulness practice

Supplement Table S11: Instrumental Variable Analysis of the teacher outcomes following the teacher training and delivery of the student MT curriculum, with allocated group an instrument for informal mindfulness practice

Supplement Table S12: Main comparisons of teacher outcomes at post-personal mindfulness training follow-up: complete case analysis

Supplement Table S13: Main comparisons of teacher outcomes at post-SBMT teacher training follow-up: complete case analysis

Supplement Table S14: Main comparisons of teacher outcomes at 1 year follow-up: complete case analysis

Supplement A. Teaching as usual: social-emotional learning

In the UK, delivering Social Emotional Learning (SEL) is rarely mandatory, and thus there is a wide variation across schools in terms of delivery. Nevertheless, SEL in the UK is usually taught as part of 'Personal, Social, Health and Economic Education' (PSHE) lessons. PSHE lessons aim to help young people develop as individuals and members of families and communities, trying to provide the knowledge, understanding, attitudes and practical skills to life healthily, safely, productively, and responsibly. Recent UK Department of Education reports suggest that 60% of secondary schools offer Personal, Social, Health and Economic Education (PSHE) lessons that are 'good or more', and that this provision is offered to students aged 11–16 years (Key Stages 3 and 4) through a variety of methods including regular scheduled lessons, drop-down days, within other subjects, and in tutor/form time [1]. Determining whether schools have good PSHE provision is challenging. In cohort 1, schools were eligible for inclusion if their provision of PSHE (or equivalent) met four criteria: (1) the presence of discrete, regular, named teaching time for PSHE, (2) a named PSHE lead, (3) a written PSHE policy, and (4) a named member of the senior leadership team responsible for PSHE.[2] However, for cohort 2, the 'written SEL policy' criterion was modified to "documentation denoting clear strategic planning of SEL within the school." Experience in cohort 1 indicated that schools do not always use the term 'SEL policy' to denote strategic planning of SEL. Moreover, in some cases, there are schools that have an extensive, well-established, and well-documented SEL curriculum, indicative of a clear structure and strategy around SEL, but do not have this formalised as a school policy [3]. While SEL provision was not uniform in the TAU arm, content is intended to prepare students with the knowledge, skills and attributes they need to manage their lives. It typically covers relationships, sex education, and physical and mental health education. TAU schools agreed not to provide the MT programme (or other curricula that include MT) until study completion.

Supplement B. School and teacher measures

School Measures

Broad context, community, and operational features

We gathered school characteristics such as the region (England, Scotland, Wales, Northern Ireland), urbanity (urban, rural), school size (<1,000 pupils, ≥1,000 pupils), type of school (mixed, girls only), Ofsted school quality rating (does not require improvement: outstanding, good; requires improvement: requires improvement, inadequate), school deprivation (% of pupils eligible for free school meals). We also described the provision quality of 'Personal, Social, Health and Economic Education' (PSHE) (see next paragraph).

School quality is measured differently in public and private schools and across the nations. We developed a measure to allow all the different school inspection rating systems to map onto each other to allow us to use the terms "outstanding" to "requires improvement."

Social Emotional Learning (SEL) in England is taught as part of 'Personal, Social, Health and Economic Education' (PSHE) lessons. Due to the fact that delivering PSHE lessons in schools is not mandatory in England, there is a wide variation across schools in the delivery of PSHE lessons (in terms of content covered and teaching time allocated). A literature review highlighted that there were no existing measures of PSHE which would allow the current study to assess which schools had a minimum level of good practice in PSHE to be considered for participation in the study. Thus, a new PSHE assessment tool was devised for the current study. For inclusion in the trial, schools had to meet 5 criteria for their current PSHE provision: regular, discrete, named teaching time for PSHE (or equivalent); a designated PSHE lead; a named member of the Senior Leadership Team (SLT) responsible for PSHE; documentation denoting clear strategic planning of SEL within the school; and evaluation of pupil progress in PSHE. Once schools became a participating trial school, PSHE was assessed by discussing PSHE provision with the teacher responsible for PSHE at each school (or a member of the Senior Leadership Team). Sixteen quality indicators (listed below) were used to assess PSHE provision. They were created specifically for this trial and identified through a review of existing measures and via expert consultation.[1] Schools were assigned a score (out of 16) reflecting the number of quality indicators present (subscale scores indicate quality in the domains of Leadership and Strategic Approaches to PSHE, Curriculum Content and Delivery and Assessment, Evaluation, and Consultation). Total scores were used in the present study. The items used, organised by sub-scales, were the following:

Sub-scales	Indicators (score)
Leadership and Strategic Approaches to PSHE from Consensus Indicators	A designated PSHE lead (0 = no, 1 = yes)<br><br>A named member of SLT has responsibility for supporting PSHE (0 = no, 1 = yes)<br><br>A written PSHE policy (0 = no, 1 = yes)<br><br>School's own rating of the quality of its PSHE provision (0-4 = 0, 5-10 = 1)<br><br>PSHE provision is part of the school improvement plan (0 = no, 1 = yes)<br><br>How well-informed does PSHE lead feel about local PSHE education CPD opportunities (0-4 = 0, 5-10 = 1)
Curriculum Content and Delivery from Consensus Indicators	Regular discrete, named teaching time for PSHE, including drop down days or tutorial time (0 = no, 1 = yes)<br><br>PSHE lead teaches PSHE lessons (0 = no, 1 = yes)<br><br>Topic Coverage KS3 and KS4 - School provides coverage of all elements of PSHE curriculum (0 = no, 1 = yes)<br><br>PSHE lead involved in planning: evidence of attempts to plan and coordinate PSHE across KS3 and KS4 (0 = no, 1 = yes)

	Teaching Methods Used: School uses at least 6/10 methods for delivering PSHE (0 = no, 1 = yes)
Methods of Assessment, Evaluation and Consultation from Consensus Indicators	Any evaluation of pupil progress in PSHE (0 = no, 1 = yes)<br><br>Informal feedback (0 = no, 1 = yes)<br><br>Pupil / peer assessment of feedback (0 = no, 1 = yes)<br><br>Written feedback on pupil progress reports (0 = no, 1 = yes)<br><br>School uses feedback to plan PSHE (0 = no, 1 = yes)

Teacher Measures

Teacher characteristics

Teacher demographic characteristics were collected at T0 via self-report questionnaires. These variables included date of birth, gender (female, male, other, prefer not to say), ethnicity (White, Asian/British Asian, Black/African/Caribbean, Mixed/Multiple Ethnic Group, Arab/Arab British, Other Ethnic Group), marital status, and mindfulness experience (currently practicing mindfulness, some understanding of mindfulness, not aware of mindfulness). We also collected information on participants' teaching experience, including qualified teaching status (yes, no), year obtained qualified teaching status, year started teaching, number of years in current school, and number of schools employed at in total.

School Climate and Connectedness Survey (SCCS)

The "School Climate and Connectedness Survey" Boards [4] measures aspects of school climate and connectedness for students, as well as aspects of school climate for teachers. From the 8-subscale measure, 3 subscales, consisting of 18 questions, were selected given their relevance to the study aims, using a 5-point Likert-type scale (from 1, "Strongly Agree", to 5, "Strongly Disagree"). These subscales assessed 'School leadership and Involvement' (e.g., "At school, decisions are made based on what is best for students", with an internal consistency in the present study of α = 0.88 at T0, being of α = 0.91 at T1, of α = 0.92 at T2, and of α = 0.92 at T3), 'Staff Attitudes' (e.g., "Teachers and staff believe that all students can do good work", with an internal consistency in the present study of α = 0.81 at T0, being of α = 0.82 at T1, of α = 0.83 at T2, and of α = 0.83 at T3), and 'Respectful Climate' (e.g., "Teachers and students treat each other with respect in this school", with an internal consistency in the present study of α = 0.84 at T0, being of α = 0.85 at T1, of α = 0.86 at T2, and of α = 0.84 at T3). Each subscale score is an average of responses across all items listed for that subscale. Thus, the higher the subscale score, the better the school climate/connectedness in that area. The mean of the 3 subscales is then taken as a total score. The Cronbach's alpha value of the SCCS total score in our study at T0 was α = 0.90, being of α = 0.91 at T1, of α = 0.92 at T2, and of α = 0.92 at T3.

Maslach Burnout Inventory Educators Survey (MBI-ES)

The "Maslach Burnout Inventory Educators Survey" [5] is a survey that was specifically designed for teachers working in an educational setting. This questionnaire determines how teachers view their job and their reactions to their work pertaining to how often they experience various aspects of occupational burnout, including feelings about students, work, and success. There are 22 items that form 3 scales: 'Emotional Exhaustion' (e.g., "I feel emotionally drained from my work", with an internal consistency in the present study of α = 0.91 at T0, T1,T2, and T3), 'Depersonalization' (e.g., "I feel I treat some students as if they were impersonal objects", with an internal consistency in the present study of α = 0.63 at T0, being of α = 0.71 at T1, of α = 0.72 at T2, and of α = 068 at T3), and (lack of) 'Personal Accomplishment' (e.g., "I deal very effectively with the problems of my students", item reversed, with an internal consistency in the present study of α = 0.75 at T0, being of α = 0.79 at T1, of α = 0.80 at T2, and of α = 0.80 at T3). Participants respond to a 7-point Likert-type scale according to the frequency the individual identifies with each statement (from 0, "Never", to 6, "Every day"). Mean scores are used to calculate each scale. We reversed the 'Personal Accomplishment' items so that higher scores in the three scales indicate higher presence of burnout symptoms. The mean of all items is used to calculate the total score. This scale has been validated for use among teaching populations.[6,7] Internal consistency of the MBI total score in our study at T0 was α = 0.88, being of α = 0.90 at T1, of α = 0.90 at T2, and of α = 0.89 at T3.

Mindfulness in Teaching Scale (MTS)

The "Interpersonal Mindfulness in Teaching Scale"[8] is a 14-item scale, assessing the ways in which mindfulness manifests in teachers with a specific focus on teachers' present-centred awareness and regulation considering the last month of teaching. The measure consists of 14 descriptive statements, and there are two subscales, 'interpersonal mindfulness' (5 items, e.g., "I listen carefully to my student's ideas, even when I disagree with them"), and 'intrapersonal mindfulness' (9 items, e.g., "I am aware of how my moods affect the way I treat my students"). Nine items are reverse scored, and the total score for each subscale is the sum of each subscale's items. Participants respond on a six-point Likert-type scale (expanded for the current study); including the original 5-point response scale (from 1, "Never True", to 5, "Always True") and an additional sixth response option included for this study (6, "Prefer Not to Say"). The IMTS has good test-retest reliability and internal consistency.[8] The internal consistency for the interpersonal mindfulness scale in the current study was $\alpha = 0.62$ at T0, being of $\alpha = 0.61$ at T1, of $\alpha = 0.63$ T2, and of $\alpha = 0.66$ at T3. The internal consistency for the intrapersonal mindfulness scale in the current study was $\alpha = 0.83$ at T0, being of $\alpha = 0.84$ at T1, of $\alpha = 0.85$ at T2, and of $\alpha = 0.84$ at T3.

Perceived Stress Scale (PSS)

The "Perceived Stress Scale"[9] is a 10-item self-report questionnaire that measures perceived stress. Questions are asked over the last month (e.g., "e.g., In the last month, how often have you been able to control irritations in your life?"), and they are measured on a 5-point Likert-type scale (from 0, "Never" to 4, "Very Often"). The PSS is reverse scored for 4 items, and the total score is calculated as the sum of all items. Scores of 20 or more are normally considered high and a score of around 13 is considered average. Internal reliability for the PSS in the current sample was $\alpha = 0.90$ at T0, being of $\alpha = 0.90$ at T1, of $\alpha = 0.91$ at T2, and of $\alpha = 0.91$ at T3.

Patient Health Questionnaire (PHQ-9)

The "Patient Health Questionnaire"[10] is a measure used to assess presence and severity of depression in the adult population. There are nine descriptive statements (e.g., "I have been feeling down, depressed or hopeless"), participants respond on a 4-point Likert-type scale (from 0, "Not at all", to 3 "Nearly every day"). The PHQ-9 also includes a tenth question to gather a global rating of functional impairment related to symptomatology ("If you checked off any problems, how difficult have these problems made it for you to do your work, take care of things at home, or get along with other people?"; participants respond on a scale from 0, "Not difficult at all" to 4, "Extremely difficult"). The PHQ-9 has excellent internal and test-retest reliability, and has demonstrated good concurrent validity, sensitivity and specificity when compared to other measures of depression.[11] Internal consistency for the PHQ-9 in the current sample was $\alpha = 0.84$ at T0, being of $\alpha = 0.81$ at T1, of $\alpha = 0.84$ at T2, and of $\alpha = 0.84$ at T3.

Generalized Anxiety Disorder-7 (GAD-7)

The "Generalized Anxiety Disorder-7"[12] is a self-report 7-item anxiety measure designed to identify cases of Generalised Anxiety Disorder. Participants respond to descriptive statements about the last two weeks (e.g. "Worrying too much about different things") on a 4-point Likert-type scale (from 0, "Not at all", to 3, "Nearly every day"). GAD-7 also includes an eighth question to assess global impact of any difficulties encountered ("If you checked off any problems, how difficult have these problems made it for you to do your work, take care of things at home, or get along with other people?") with responses from 0, "Not difficult at all" to 4, "Extremely difficult". The GAD-7 has strong criterion and construct validity, and a one factor structure.[13] A score of 10 or greater on the GAD-7 represents a reasonable cut-off point for identifying cases of GAD. Cut-off points of 5, 10, and 15 can also be interpreted as representing mild, moderate, and severe levels of anxiety on the GAD-7. The internal consistency for the GAD-7 in the current sample is $\alpha = 0.89$ at T0, being of $\alpha = 0.87$ at T1, of $\alpha = 0.89$ at T2, and of $\alpha = 0.90$ at T3.

Five Facet Mindfulness Questionnaire, Short Form (FFMQ-SF)

The "Five Facet Mindfulness Questionnaire Short Form"[14] is a 24-item short form version of the 39-item Five Facet Mindfulness Questionnaire.[15,16] The FFMQ-SF is a questionnaire used to assess different components of mindfulness in clinical, community, and student populations. The questionnaire contains the following five

subscales: ‘non-react’ (e.g., “I watch my feelings without getting carried away by them”, with an internal consistency in the present study of α = 0.74 at T0, being of α = 0.76 at T1, of α = 0.76 at T2, and of α = 0.78 at T3), ‘observe’ (e.g., “I pay attention to physical experiences, such as the wind in my hair or sun on my face”, with an internal consistency in the present study of α = 0.80 at T0, being of α = 0.85 at T1, of α = 0.86 at T2, and of α = 0.87 at T3), ‘act aware’ (e.g., “I do jobs or tasks automatically without being aware of what I’m doing”, item reversed, with an internal consistency in the present study of α = 0.87 T0, being of α = 0.88 at T1, of α = 0.88 at T2, and of α = 0.89 at T3), ‘describe’ (e.g., “It’s hard for me to find the words to describe what I’m thinking”, item reversed, with an internal consistency in the present study of α = 0.85 at T0, being of α = 0.87 at T1, of α = 0.87 at T2, and of α = 0.83 at T3) and ‘non-judge’ (e.g., “I think some of my emotions are bad or inappropriate and I shouldn’t feel them”, item reversed, with an internal consistency in the present study of α = 0.77 at T0, being of α = 0.76 at T1, of α = 0.80 at T2, and of α = 0.81 at T3). Participants responded on a 5-point Likert-type scale from 1, “Never or very rarely true”, to 5 “Very often or always true”, about how frequently they had experienced each item in the last month. Total scores were calculated using overall sum scores for the subscales, with 12 items reverse scored. The higher the score, the higher the participants self-reported level of mindfulness skills. The internal consistency for the FFMQ-SF total score in the current sample was α = 0.88 at T0, being of α = 0.89 at T1, of α = 0.91 at T2, and of α = 0.90 at T3.

Teachers' Sense of Efficacy Scale (TSES)

The “Teachers' Sense of Efficacy Scale”[17] assesses teachers’ sense of self-efficacy. The TSES scale is either a 24-item long form or 12-item short form questionnaire. The present study used the 12-item short form,[18] as the longer scale is deemed most suitable for pre-service teachers, which was not relevant in our study. The TSES scale measures the following three components: ‘efficacy for instructional strategies’ (e.g., “To what extent can you craft good questions for your students?”, with an internal consistency in the present study of α = 0.75 at T0, being of α = 0.77 at T1, of α = 0.81 at T2, and of α = 0.76 at T3), ‘efficacy for classroom management’ (e.g., “How much can you do to control disruptive behaviour in the classroom?”, with an internal consistency in the present study of α = 0.86 at T0, being of α = 0.85 at T1, of α = 0.88 at T2, and of α = 0.85 at T3), and ‘efficacy for student engagement’ (e.g., “How much can you do to motivate students who show low interest in school work?”, with an internal consistency in the present study of α = 0.76 at T0, being of α = 0.78 at T1, of α = 0.81 at T2, and of α = 0.80 at T3). Participants are asked to respond to each item by considering their current capabilities using a 9-point Likert-type scale ranging from 1, “None at all” to 9, “A great deal”. Scores are calculated using the mean of all the corresponding items. This scale has been validated for use among teacher populations.[18] The internal consistency for the TSES total score in the current sample was α = 0.87 at T0, being of α = 0.89 at T1, of α = 0.90 at T2, and of α = 0.88 at T3.

Engagement with the SBMT

We assessed the participating teachers’ engagement with the SBMT in two ways. First their personal MT was assessed through two self-report items asking about the frequency of formal (structured, scheduled) and informal (everyday, flexible) mindfulness practices over the last month, asked at two time points, following the MT and at follow-up. Questions asked were as follows: “In the last month on average, did you complete at least one formal mindfulness practice (e.g., a guided meditation such as the body scan, breath & body, sounds and thoughts, mindful movement etc.)”, and “In the last month on average, did you complete some informal mindfulness practice (e.g. bringing attention to daily activities, ‘keeping the body in mind’ when teaching, taking an unscheduled breathing space”). Responses were provided on a 4-point Likert-type scale, with 0 (“no practice”), 1 (“at least occasionally”), 2 (“at least several times a week”) and 3 (“yes, daily”). Second, the extent to which participating teachers completed the full training/implementation route of the SBMT was recorded. The response set was: (1) not completed any training, (2) completed only personal MT (at least 4 of 8 MBCT-L sessions), (3) completed the SBMT teacher training and then went on to teach mindfulness to students.

Supplement C. Representativeness of study schools

Eighty seven percent of schools were mixed (the remainder being girls only schools); 13% required improvement based on their school quality rating (for schools outside England, they were reviewed and scored as per the England ratings to align to the same scale); and just over a third of schools had a higher percentage of children eligible for free school meals than the national median (29.4%).[19]

Supplementary Table S1: Selected teacher baseline characteristics at post-personal MT (MBCT-L) and SBMT teacher training follow-up for remaining teachers and teachers lost to follow up

Variables	Teachers lost to follow-up*			Remaining teachers**		
	**Intervention (N = 93)**	**Control (N = 95)**	**Total (N = 188)**	**Intervention (N = 269)**	**Control (N = 222)**	**Total (N = 491)**
Age†, mean (SD)	39.3 (10.1)	38.1 (9.3)	38.7 (9.7)	40.5 (8.4)	39.6 (9.2)	40.1 (8.8)
Gender						
Female, n (%)	68 (73.9)	68 (71.6)	136 (72.7)	215 (79.9)	155 (69.8)	370 (75.4)
Male, n (%)	24 (26.1)	27 (28.4)	51 (27.3)	53 (19.7)	66 (29.7)	119 (24.2)
Other, n (%)	0 (0.0)	0 (0.0)	0 (0.0)	1 (0.4)	1 (0.5)	2 (0.4)
Ethnicity						
Arab/Arab British, n (%)	0 (0.0)	0 (0.0)	0 (0.0)	0 (0.0)	2 (0.9)	2 (0.4)
Asian/British Asian, n (%)	3 (3.3)	7 (7.4)	10 (5.4)	4 (1.5)	6 (2.7)	10 (2.0)
Black/African/Caribbean, n (%)	1 (1.1)	1 (1.1)	2 (1.1)	1 (0.4)	2 (0.9)	3 (0.6)
Mixed/Multiple Ethnic Group, n (%)	3 (3.3)	2 (2.1)	5 (2.7)	6 (2.2)	2 (0.9)	8 (1.6)
White, n (%)	85 (92.4)	84 (88.4)	169 (90.4)	256 (95.2)	208 (93.7)	464 (94.5)
Other Ethnic Group, n (%)	0 (0.0)	1 (1.1)	1 (0.5)	2 (0.7)	2 (0.9)	4 (0.8)
Qualified teacher status††, n (%)	91 (98.9)	94 (99.0)	185 (98.9)	262 (97.4)	215 (96.9)	477 (97.2)
Years teaching experience†††, mean (SD)	10.6 (8.3)	11.8 (8.2)	11.2 (8.3)	13.7 (8.4)	12.4 (8.8)	13.1 (8.6)

* Defined as those teachers with missing data on all outcomes at pre-intervention.
** Defined as those teachers with at least one of the outcomes at pre-intervention.
† Sample size in teachers lost to follow up group: 186: intervention arm: 92; control arm: 94.
†† 92 teachers in intervention arm of lost to follow up group provided data on qualified teacher status.
††† 92 teachers in intervention arm of lost to follow up group provided data on years teaching experience.

Supplementary Table S2: Selected teacher baseline characteristics at post MT (MBCT-L) and SBMT teacher training for remaining teachers and teachers lost to follow up

**Variables**	**Teachers lost to follow-up***			**Remaining teachers****		
	**Intervention (N = 153)**	**Control (N = 132)**	**Total (N = 285)**	**Intervention (N = 209)**	**Control (N = 185)**	**Total (N = 394)**
Age†, mean (SD)	38.4 (9.2)	37.8 (8.8)	38.1 (9.0)	41.5 (8.4)	40.1 (9.4)	40.8 (8.9)
Gender						
Female, n (%)	116 (76.3)	95 (72.0)	211 (74.3)	167 (79.9)	128 (69.2)	295 (74.9)
Male, n (%)	36 (23.7)	37 (28.0)	73 (25.7)	41 (19.6)	56 (30.3)	97 (24.6)
Other, n (%)	0 (0.0)	0 (0.0)	0 (0.0)	1 (0.5)	1 (0.5)	2 (0.5)
Ethnicity						
Arab/Arab British, n (%)	0 (0.0)	1 (0.8)	1 (0.4)	0 (0.0)	1 (0.5)	1 (0.3)
Asian/British Asian, n (%)	4 (2.6)	9 (6.8)	13 (4.6)	3 (1.4)	4 (2.2)	7 (1.8)
Black/African/Caribbean, n (%)	2 (1.3)	2 (1.5)	4 (1.4)	0 (0.0)	1 (0.5)	1 (0.3)
Mixed/Multiple Ethnic Group, n (%)	6 (4.0)	2 (1.5)	8 (2.8)	3 (1.4)	2 (1.1)	5 (1.3)
White, n (%)	140 (92.1)	116 (87.9)	256 (90.1)	201 (96.2)	176 (95.1)	377 (95.7)
Other Ethnic Group, n (%)	0 (0.0)	2 (1.5)	2 (0.7)	2 (1.0)	1 (0.5)	3 (0.8)
Qualified teacher status††, n (%)	151 (99.3)	130 (98.5)	281 (98.9)	202 (96.7)	179 (96.8)	381 (96.7)
Years teaching experience†††, mean (SD)	10.9 (8.3)	11.5 (7.9)	11.2 (8.1)	14.4 (8.4)	12.7 (9.1)	13.6 (8.8)

* Defined as those teachers with missing data on all outcomes at post-intervention.
** Defined as those teachers with at least one of the outcomes at post-intervention.
† Sample size in teachers lost to follow up group: 283: intervention arm: 152; control arm: 131.
†† 152 teachers in intervention arm of lost to follow up group provided data on qualified teacher status.
††† 152 teachers in intervention arm of lost to follow up group provided data on years teaching experience.

Supplementary Table S3: Selected teachers baseline characteristics at 1 year follow-up status for remaining teachers and teachers lost to follow up

**Variables**	**Teachers lost to follow-up***			**Remaining teachers****		
	**Intervention (N = 148)**	**Control (N = 145)**	**Total (N = 293)**	**Intervention (N = 214)**	**Control (N = 172)**	**Total (N = 386)**
Age[†], mean (SD)	39.8 (9.3)	38.0 (9.0)	38.9 (9.2)	40.5 (8.6)	40.1 (9.3)	40.3 (8.9)
Gender						
Female, n (%)	114 (77.6)	103 (71.0)	217 (74.3)	169 (79.0)	120 (69.8)	289 (74.9)
Male, n (%)	33 (22.5)	41 (28.3)	74 (25.3)	44 (20.6)	52 (30.2)	96 (24.9)
Other, n (%)	0 (0.0)	1 (0.7)	1 (0.3)	1 (0.5)	0 (0.0)	1 (0.3)
Ethnicity						
Arab/Arab British, n (%)	0 (0.0)	1 (0.7)	1 (0.3)	0 (0.0)	1 (0.6)	1 (0.3)
Asian/British Asian, n (%)	4 (2.7)	9 (6.2)	13 (4.5)	3 (1.4)	4 (2.3)	7 (1.8)
Black/African/Caribbean, n (%)	2 (1.4)	1 (0.7)	3 (1.0)	0 (0.0)	2 (1.2)	2 (0.5)
Mixed/Multiple Ethnic Group, n (%)	5 (3.4)	2 (1.4)	7 (2.4)	4 (1.9)	2 (1.2)	6 (1.6)
White, n (%)	136 (92.5)	130 (89.7)	266 (91.1)	205 (95.8)	162 (94.2)	367 (95.1)
Other Ethnic Group, n (%)	0 (0.0)	2 (1.4)	2 (0.7)	2 (0.9)	1 (0.6)	3 (0.8)
Qualified teacher status[††], n (%)	146 (99.3)	143 (98.6)	289 (99.0)	207 (96.7)	166 (96.5)	373 (96.6)
Years teaching experience[†††], mean (SD)	11.5 (8.6)	12.0 (8.5)	11.7 (8.6)	14.0 (8.3)	12.4 (8.7)	13.3 (8.5)

* Defined as those teachers with missing data on all outcomes at 1 year.
** Defined as those teachers with at least one of the outcomes at 1 year.
[†] Sample size in teachers lost to follow up group: 291: intervention arm: 147; control arm: 144.
[††] 147 teachers in intervention arm of lost to follow up group provided data on qualified teacher status.
[†††] 147 teachers in intervention arm of lost to follow up group provided data on years teaching experience.

Supplementary Table S4: Main comparisons of teacher outcomes following personal MT (MBCT-L) and SBMT teacher training, but before delivery of the student MT in the SBMT arm, and at comparable timepoint in TAU arm.

**Outcome**	**SBMT arm (I)**		**TAU arm (C)**		**Unadjusted mean difference**	**Adjusted mean difference**			**ICC**[a]
	**N**	**mean (SD)**	**N**	**mean (SD)**	**estimate**	**estimate**	**95% CI**	**p-value**	
**Burn out – Maslach Burn-Out Inventory (MBI)**									
Exhaustion									
ITT	264	2.5 (1.2)	221	2.5 (1.3)	-0.1	-0.2	-0.3 to 0.01	0.066	0.019
CACE (i)[b]					-0.2	-0.2	-0.4 to 0.0	0.050	-
Depersonalisation									
ITT	264	0.7 (0.8)	221	0.8 (1.0)	-0.1	-0.1	-0.2 to 0.1	0.367	0.139
CACE (i)[b]					-0.1	-0.1	-0.3 to 0.1	0.379	-
Personal Accomplishment									
ITT	264	1.0 (0.7)	221	1.1 (0.8)	-0.1	-0.1	-0.2 to 0.03	0.159	0.061
CACE (i)[b]					-0.1	-0.1	-0.3 to 0.04	0.132	-
**Self-efficacy Questionnaire (TSES)**									
Student Engagement Subscale									
ITT	258	7.0 (1.0)	215	6.7 (1.2)	0.3	0.2	0.04 to 0.4	0.017	0.068

CACE (i)[b]	231	6.9 (1.1)	215	6.7 (1.2)	0.4	0.3	0.1 to 0.5	0.013	-
Instructional Practice Subscale									
ITT	258	7.4 (0.9)	215	7.4 (1.0)	-0.01	0.02	-0.2 to 0.2	0.792	0.068
CACE (i)[b]					-0.01	0.03	-0.2 to 0.2	0.797	-
Classroom Management Subscale									
ITT	258	7.7 (0.8)	215	7.6 (1.0)	0.2	0.1	-0.04 to 0.2	0.143	0.053
CACE (i)[b]					0.2	0.1	-0.05 to 0.3	0.148	-
**Mindfulness (FFMQ-SF)**									
ITT	260	84.7 (12.6)	215	85.4 (13.1)	-1.2	-0.4	-2.3 to 1.6	0.706	0.035
CACE (i)[b]					-1.7	-0.4	-2.9 to 2.0	0.721	-
**Mindfulness (MTS) - Interpersonal**									
ITT	262	20.7 (2.4)	216	20.7 (2.7)	-0.1	-0.1	-0.5 to 0.3	0.726	0.035
CACE (i)[b]					-0.1	-0.1	-0.6 to 0.5	0.734	-
**Mindfulness (MTS) - Intrapersonal**									
ITT	262	32.0 (4.9)	217	34.0 (5.7)	-1.8	-1.4	-2.2 to -0.6	<0.001	0.053
CACE (i)[b]					-2.4	-1.8	-2.8 to -0.8	<0.001	-
**Stress (PSS)**									
ITT	262	16.2 (7.0)	217	15.8 (7.1)	0.2	0.4	-0.6 to 1.5	0.402	0.050
CACE (i)[b]					0.4	0.6	-0.7 to 1.9	0.358	-

**Depression (PHQ-9)**									
ITT	262	5.1 (3.9)	217	4.7 (4.0)	0.3	0.4	-0.2 to 1.0	0.233	0.029
CACE (i)[b]					0.4	0.5	-0.3 to 1.3	0.200	-
**Anxiety (GAD7)**									
ITT	261	4.3 (3.6)	216	4.4 (4.0)	-0.2	0.04	-0.6 to 0.6	0.906	0.037
CACE (i)[b]					-0.3	0.03	-0.7 to 0.8	0.938	-
School ecology/climate (SCCS)									
School leadership and involvement									
ITT	269	3.9 (0.7)	222	3.8 (0.7)	0.1	0.2	0.04 to 0.3	0.011	0.174
CACE (i)[b]					0.2	0.2	0.1 to 0.4	0.006	-
Staff attitudes									
ITT	269	4.1 (0.6)	222	4.1 (0.5)	-0.1	-0.01	-0.1 to 0.1	0.902	0.232
CACE (i)[b]					-0.1	-0.003	-0.1 to 0.1	0.963	-
Respectful Climate									
ITT	269	3.8 (0.6)	222	3.7 (0.6)	0.1	0.1	-0.01 to 0.2	0.087	0.257
CACE (i)[b]					0.1	0.1	-0.01 to 0.2	0.066	-

[a] Intra-cluster (intra-school) correlation coefficients (ICCs) from crude (unadjusted) analyses.
[b] A participant in the intervention arm is deemed a complier if they have attended at least four of the eight personal mindfulness sessions.

Supplementary Table S5: Main comparisons of teacher outcomes post personal MT (MBCT-L) and SBMT teacher training and delivery of the pupil MT

**Outcome**	**SBMT arm (I)**		**TAU arm (C)**		**Unadjusted mean Difference (I-C)**	**Adjusted mean difference (I-C)**			**ICC[a]**
	**N**	**mean (SD)**	**N**	**mean (SD)**	**estimate**	**estimate**	**95% CI**	**p-value**	
**Well-being – Maslach Burn-Out Inventory (MBI)**									
Exhaustion									
ITT	206	2.3 (1.1)	181	2.5 (1.3)	-0.2	-0.3	-0.5 to -0.1	0.010	0.043
CACE (i)[b]					-0.3	-0.4	-0.6 to -0.1	0.009	-
CACE (ii)[c]					-0.7	-0.8	-1.3 to -0.2	0.006	-
Depersonalisation									
ITT	206	0.8 (0.8)	181	0.9 (1.0)	-0.2	-0.2	-0.3 to 0.01	0.074	0.072
CACE (i)[b]					-0.3	-0.2	-0.4 to -0.001	0.049	-
CACE (ii)[c]					-0.5	-0.5	-0.9 to -0.1	0.024	-
Personal Accomplishment									
ITT	206	1.0 (0.7)	181	1.1 (0.8)	-0.2	-0.2	-0.3 to -0.01	0.034	0.045
CACE (i)[b]					-0.2	-0.2	-0.4 to -0.03	0.025	-

CACE (ii)[c]					-0.5	-0.5	-0.9 to -0.1	0.013	-
**Self-efficacy Questionnaire (TSES)**									
Student Engagement Subscale									
ITT	197	6.9 (1.1)	174	6.7 (1.2)	0.1	0.05	-0.2 to 0.3	0.677	0.117
CACE (i)[b]					0.1	0.1	-0.2 to 0.3	0.677	-
CACE (ii)[c]					0.3	0.2	-0.4 to 0.8	0.560	-
Instructional Practice Subscale									
ITT	197	7.5 (1.0)	174	7.4 (1.1)	0.1	0.1	-0.1 to 0.3	0.258	0.099
CACE (i)[b]					0.1	0.1	-0.1 to 0.4	0.266	-
CACE (ii)[c]					0.2	0.3	-0.2 to 0.9	0.196	-
Classroom Management Subscale									
ITT	197	7.7 (0.9)	174	7.5 (1.1)	0.1	0.04	-0.1 to 0.2	0.662	0.093
CACE (i)[b]					0.1	0.0	-0.2 to 0.3	0.678	-
CACE (ii)[c]					0.2	0.3	-0.3 to 0.6	0.587	-
**Mindfulness (FFMQ-SF)**									
ITT	199	86.6 (13.6)	174	85.8 (13.7)	0.5	1.3	-0.8 to 3.5	0.230	0.018
CACE (i)[b]					0.6	1.9	-0.9 to 4.7	0.183	-
CACE (ii)[c]					1.3	4.9	-0.7 to 10.5	0.089	-
**Mindfulness (MTS) - Interpersonal**									

ITT	201	20.8 (2.3)	176	20.5 (2.8)	0.3	0.2	-0.2 to 0.7	0.305	0.047
CACE (i)[b]					0.4	0.3	-0.3 to 1.0	0.265	-
CACE (ii)[c]					0.8	1.0	-0.3 to 2.3	0.125	-
**Mindfulness (MTS) - Intrapersonal**									
ITT	200	32.1 (5.1)	176	33.8 (5.5)	-1.2	-0.8	-1.6 to 0.1	0.067	0.056
CACE (i)[b]					-1.6	-1.0	-2.1 to 0.1	0.075	-
CACE (ii)[c]					-3.5	-1.7	-3.9 to 0.5	0.127	-
**Stress (PSS)**									
ITT	201	14.6 (6.5)	175	15.5 (7.4)	-0.9	-0.8	-2.0 to 0.3	0.166	0.048
CACE (i)[b]					-1.2	-1.1	-2.6 to 0.4	0.146	-
CACE (ii)[c]					-2.6	-2.3	-5.3 to 0.7	0.137	-
**Depression (PHQ-9)**									
ITT	200	4.7 (3.8)	174	5.1 (4.6)	-0.4	-0.3	-1.0 to 0.4	0.380	0.030
CACE (i)[b]					-0.5	-0.4	-1.3 to 0.4	0.320	-
CACE (ii)[c]					-1.2	-0.9	-2.6 to 0.7	0.270	-
**Anxiety (GAD7)**									
ITT	200	4.0 (3.6)	174	4.6 (4.1)	-0.7	-0.5	-1.3 to 0.2	0.142	0.036
CACE (i)[b]					-0.9	-0.8	-1.7 to 0.2	0.104	-
CACE (ii)[c]					-2.0	-1.7	-3.5 to 0.2	0.075	-

School ecology/climate (SCCS)									
School leadership and involvement									
ITT	209	3.9 (0.7)	185	3.6 (0.8)	0.2	0.2	0.03 to 0.3	0.019	0.211
CACE (i)[b]					0.2	0.3	0.1 to 0.5	0.007	-
CACE (ii)[c]					0.5	0.6	0.2 to 1.0	0.006	-
Staff attitudes									
ITT	209	4.1 (0.6)	185	4.0 (0.6)	0.04	0.1	-0.02 to 0.2	0.111	0.268
CACE (i)[b]					0.1	0.2	0.004 to 0.3	0.044	-
CACE (ii)[c]					0.2	0.3	0.03 to 0.6	0.031	-
Respectful Climate									
ITT	209	3.8 (0.5)	185	3.6 (0.6)	0.2	0.2	0.03 to 0.3	0.012	0.309
CACE (i)[b]					0.2	0.2	0.1 to 0.4	0.009	-
CACE (ii)[c]					0.4	0.4	0.1 to 0.7	0.012	-

[a] Intra-cluster (intra-school) correlation coefficients (ICCs) from crude (unadjusted) analyses.

[b] A participant in the intervention arm is deemed a complier if they have attended at least four of the eight personal mindfulness sessions.

[c] A participant in the intervention arm is deemed a complier if they have attended at least four of the eight personal mindfulness sessions and the four-day training course, and have delivered at least one mindfulness session.

Supplementary Table S6: Instrumental Variable Analysis of the teacher outcomes at 1-year follow-up, with allocated group as an instrument for formal mindfulness practice

**Outcome**	**SBMT arm (I)**		**TAU arm (C)**		**Unadjusted mean Difference (I-C)**	**Adjusted mean difference (I-C)**		
	**N**	**mean (SD)**	**N**	**mean (SD)**	**estimate**	**estimate**	**95% CI**	**p-value**
**Burnout – Maslach Burnout Inventory (MBI)**								
Exhaustion								
ITT	210	2.4 (1.2)	171	2.5 (1.2)	-0.1	-0.1	-0.3 to 0.1	0.247
Cutoff One[a]					-0.2	-0.3	-0.6 to 0.04	0.088
Cutoff Two[b]					-0.5	-0.6	-1.7 to 0.4	0.224
Cutoff Three[c]					-3.9	-2.5	-9.3 to 4.2	0.458
Depersonalisation								
ITT	210	0.7 (0.7)	171	0.9 (0.9)	-0.1	-0.1	-0.2 to 0.1	0.362
Cutoff One[a]					-0.2	-0.2	-0.4 to 0.04	0.100
Cutoff Two[b]					-0.6	-0.3	-1.1 to 0.4	0.383
Cutoff Three[c]					-4.4	-3.3	-9.3 to 2.8	0.292
Personal Accomplishment								
ITT	210	1.0 (0.7)	171	1.1 (0.8)	-0.1	-0.1	-0.2 to 0.1	0.258

Cutoff One[a]					-0.2	-0.1	-0.4 to 0.1	0.163
Cutoff Two[b]					-0.5	-0.3	-0.9 to 0.4	0.435
Cutoff Three[c]					-3.9	-1.9	-6.5 to 2.7	0.412
**Self-efficacy Questionnaire (TSES)**								
Student Engagement Subscale								
ITT	204	7.0 (1.1)	162	6.8 (1.1)	0.2	0.1	-0.1 to 0.3	0.269
Cutoff One[a]					0.2	0.2	-0.1 to 0.5	0.222
Cutoff Two[b]					0.8	0.6	-0.4 to 1.7	0.243
Cutoff Three[c]					5.9	4.9	-2.8 to 12.6	0.207
Instructional Practice Subscale								
ITT	204	7.5 (0.8)	162	7.5 (1.0)	0.001	0.03	-0.1 to 0.2	0.704
Cutoff One[a]					-0.002	0.1	-0.2 to 0.3	0.581
Cutoff Two[b]					-0.01	0.2	-0.7 to 1.0	0.684
Cutoff Three[c]					-0.1	1.6	-4.1 to 7.3	0.583
Classroom Management Subscale								
ITT	204	7.8 (0.8)	162	7.6 (0.9)	0.2	0.1	-0.04 to 0.3	0.132
Cutoff One[a]					0.3	0.3	-0.03 to 0.5	0.077
Cutoff Two[b]					1.0	0.7	-0.2 to 1.6	0.134
Cutoff Three[c]					7.3	5.2	-2.2 to 12.5	0.165

**Mindfulness (FFMQ-SF)**								
ITT	205	85.4 (12.8)	162	86.1 (13.9)	-0.8	-0.2	-2.4 to 2.0	0.854
Cutoff One[a]					-1.5	0.8	-2.6 to 4.1	0.659
Cutoff Two[b]					-5.1	1.5	-9.5 to 12.4	0.792
Cutoff Three[c]					-37.7	15.7	-57.6 to 89.1	0.672
**Mindfulness (MTS) - Interpersonal**								
ITT	208	20.8 (2.7)	168	20.9 (2.4)	-0.2	-0.2	-0.7 to 0.2	0.333
Cutoff One[a]					-0.4	-0.3	-1.0 to 0.3	0.319
Cutoff Two[b]					-1.3	-1.3	-3.3 to 0.8	0.222
Cutoff Three[c]					-9.4	-3.6	-18.5 to 11.4	0.639
**Mindfulness (MTS) - Intrapersonal**								
ITT	208	32.1 (5.0)	168	33.4 (5.6)	-1.2	-0.9	-1.7 to -0.1	0.020
Cutoff One[a]					-2.1	-1.2	-2.5 to 0.04	0.058
Cutoff Two[b]					-7.0	-5.0	-9.3 to -0.7	0.022
Cutoff Three[c]					-51.7	-28.0	-62.1 to 6.0	0.107
**Stress (PSS)**								
ITT	207	15.3 (6.9)	165	15.9 (7.4)	-0.3	0.01	-1.1 to 1.2	0.982
Cutoff One[a]					-0.3	-0.6	-2.4 to 1.3	0.531
Cutoff Two[b]					-1.1	-2.0	-8.4 to 4.5	0.547

Cutoff Three[c]					-8.1	-18.0	-63.3 to 27.4	0.434
**Depression (PHQ-9)**								
ITT	206	4.7 (4.1)	164	5.0 (4.2)	-0.3	-0.1	-1.0 to 0.7	0.731
Cutoff One[a]					-0.5	-0.3	-1.7 to 1.0	0.647
Cutoff Two[b]					-1.7	-0.4	-4.8 to 4.0	0.857
Cutoff Three[c]					-12.5	-7.7	-36.6 to 21.2	0.598
**Anxiety (GAD7)**								
ITT	206	4.2 (3.9)	163	4.3 (4.1)	-0.1	0.2	-0.7 to 1.0	0.696
Cutoff One[a]					-0.2	0.1	-1.3 to 1.5	0.874
Cutoff Two[b]					-0.6	0.7	-3.9 to 5.4	0.756
Cutoff Three[c]					-4.1	8.5	-22.9 to 40.0	0.590
School ecology/climate (SCCS)								
School leadership and involvement								
ITT	211	3.9 (0.7)	171	3.8 (0.7)	0.1	0.1	-0.04 to 0.3	0.144
Cutoff One[a]					0.2	0.2	-0.01 to 0.5	0.055
Cutoff Two[b]					0.6	0.6	-0.2 to 1.4	0.122
Cutoff Three[c]					4.6	4.0	-1.6 to 9.7	0.159
Staff attitudes								
ITT	211	4.1 (0.6)	171	4.1 (0.5)	0.03	0.1	-0.1 to 0.2	0.253

Cutoff One[a]					0.04	0.2	-0.04 to 0.4	0.109
Cutoff Two[b]					0.1	0.5	-0.1 to 1.2	0.123
Cutoff Three[c]					1.0	3.5	-1.3 to 8.3	0.152
Respectful Climate								
ITT	211	3.9 (0.5)	171	3.7 (0.6)	0.1	0.2	0.02 to 0.3	0.020
Cutoff One[a]					0.2	0.3	0.1 to 0.5	0.006
Cutoff Two[b]					0.8	0.8	0.1 to 1.5	0.020
Cutoff Three[c]					5.6	6.2	0.1 to 12.2	0.045

[a] A participant in the SBMT arm is deemed a complier if they have reported undertaking formal mindfulness practice at least occasionally.

[b] A participant in the SBMT arm is deemed a complier if they have reported undertaking formal mindfulness practice at least several times a week.

[c] A participant in the SBMT arm is deemed a complier if they have reported undertaking formal mindfulness practice daily.

Supplementary Table S7: Instrumental Variable Analysis of the teacher outcomes at 1-year follow-up, with allocated group as an instrument for informal mindfulness practice

**Outcome**	**SBMT arm (I)**		**TAU arm (C)**		**Unadjusted mean difference**	**Adjusted mean difference**		
	**N**	**mean (SD)**	**N**	**mean (SD)**	**estimate**	**estimate**	**95% CI**	**p-value**
**Well-being – Maslach Burn-Out Inventory (MBI)**								
Exhaustion								
ITT	210	2.4 (1.2)	171	2.5 (1.2)	-0.1	-0.1	-0.3 to 0.1	0.247
Cutoff One[a]					-0.1	-0.2	-0.5 to 0.1	0.138
Cutoff Two[b]					-0.3	-0.4	-0.9 to 0.1	0.101
Cutoff Three[c]					-0.7	-0.7	-2.2 to 0.8	0.383
Depersonalisation								
ITT	210	0.7 (0.7)	171	0.9 (0.9)	-0.1	-0.1	-0.2 to 0.1	0.362
Cutoff One[a]					-0.1	-0.1	-0.3 to 0.1	0.160
Cutoff Two[b]					-0.3	-0.3	-0.7 to 0.02	0.065
Cutoff Three[c]					-0.8	-0.5	-1.6 to 0.6	0.341
Personal Accomplishment								
ITT	210	1.0 (0.7)	171	1.1 (0.8)	-0.1	-0.1	-0.2 to 0.1	0.258

Cutoff One[a]					-0.1	-0.1	-0.3 to 0.1	0.169
Cutoff Two[b]					-0.3	-0.2	-0.6 to 0.1	0.154
Cutoff Three[c]					-0.7	-0.4	-1.3 to 0.5	0.401
**Self-efficacy Questionnaire (TSES)**								
Student Engagement Subscale								
ITT	204	7.0 (1.1)	162	6.8 (1.1)	0.2	0.1	-0.1 to 0.3	0.269
Cutoff One[a]					0.2	0.2	-0.1 to 0.4	0.244
Cutoff Two[b]					0.4	0.3	-0.2 to 0.9	0.193
Cutoff Three[c]					1.1	0.9	-0.6 to 2.4	0.232
Instructional Practice Subscale								
ITT	204	7.5 (0.8)	162	7.5 (1.0)	0.001	0.03	-0.1 to 0.2	0.704
Cutoff One[a]					-0.002	0.05	-0.2 to 0.3	0.650
Cutoff Two[b]					-0.003	0.1	-0.3 to 0.5	0.506
Cutoff Three[c]					-0.01	0.2	-0.9 to 1.4	0.689
Classroom Management Subscale								
ITT	204	7.8 (0.8)	162	7.6 (0.9)	0.2	0.1	-0.04 to 0.3	0.132
Cutoff One[a]					0.2	0.2	-0.04 to 0.4	0.100
Cutoff Two[b]					0.5	0.4	-0.02 to 0.9	0.062
Cutoff Three[c]					1.4	1.0	-0.3 to 2.4	0.123

**Mindfulness (FFMQ-SF)**								
ITT	205	85.4 (12.8)	162	86.1 (13.9)	-0.8	-0.2	-2.4 to 2.0	0.854
Cutoff One[a]					-1.2	0.3	-2.5 to 3.0	0.854
Cutoff Two[b]					-2.5	2.1	-3.4 to 7.5	0.454
Cutoff Three[c]					-7.0	1.6	-14.3 to 17.6	0.838
**Mindfulness (MTS) - Interpersonal**								
ITT	208	20.8 (2.7)	168	20.9 (2.4)	-0.2	-0.2	-0.7 to 0.2	0.333
Cutoff One[a]					-0.3	-0.3	-0.8 to 0.3	0.343
Cutoff Two[b]					-0.7	-0.5	-1.6 to 0.6	0.359
Cutoff Three[c]					-1.8	-1.1	-4.1 to 2.0	0.490
**Mindfulness (MTS) - Intrapersonal**								
ITT	208	32.1 (5.0)	168	33.4 (5.6)	-1.2	-0.9	-1.7 to -0.1	0.020
Cutoff One[a]					-1.7	-1.1	-2.1 to -0.04	0.041
Cutoff Two[b]					-3.6	-1.6	-3.5 to 0.4	0.119
Cutoff Three[c]					-9.8	-7.4	-13.9 to -1.0	0.024
**Stress (PSS)**								
ITT	207	15.3 (6.9)	165	15.9 (7.4)	-0.3	0.01	-1.1 to 1.2	0.982
Cutoff One[a]					-0.3	-0.3	-1.8 to 1.3	0.734
Cutoff Two[b]					-0.6	-1.3	-4.4 to 1.9	0.432

Cutoff Three[c]					-1.6	-0.9	-10.3 to 8.4	0.840
**Depression (PHQ-9)**								
ITT	206	4.7 (4.1)	164	5.0 (4.2)	-0.3	-0.1	-1.0 to 0.7	0.731
Cutoff One[a]					-0.4	-0.2	-1.3 to 0.9	0.690
Cutoff Two[b]					-0.9	-0.5	-2.7 to 1.6	0.627
Cutoff Three[c]					-2.4	-1.5	-7.9 to 4.8	0.633
**Anxiety (GAD7)**								
ITT	206	4.2 (3.9)	163	4.3 (4.1)	-0.1	0.2	-0.7 to 1.0	0.696
Cutoff One[a]					-0.1	0.2	-1.0 to 1.3	0.779
Cutoff Two[b]					-0.3	0.1	-2.1 to 2.3	0.934
Cutoff Three[c]					-0.8	1.0	-5.4 to 7.4	0.761
School ecology/climate (SCCS)								
School leadership and involvement								
ITT	211	3.9 (0.7)	171	3.8 (0.7)	0.1	0.1	-0.04 to 0.3	0.144
Cutoff One[a]					0.2	0.2	-0.02 to 0.4	0.078
Cutoff Two[b]					0.3	0.4	-0.01 to 0.8	0.058
Cutoff Three[c]					0.9	0.8	-0.4 to 2.0	0.213
Staff attitudes								
ITT	211	4.1 (0.6)	171	4.1 (0.5)	0.03	0.1	-0.1 to 0.2	0.253

BMJ Publishing Group Limited (BMJ) disclaims all liability and responsibility arising from any reliance placed on this supplemental material which has been supplied by the author(s)

Cutoff One[a]					0.03	0.1	-0.05 to 0.3	0.160
Cutoff Two[b]					0.1	0.3	-0.05 to 0.6	0.093
Cutoff Three[c]					0.2	0.5	-0.4 to 1.5	0.234
Respectful Climate								
ITT	211	3.9 (0.5)	171	3.7 (0.6)	0.1	0.2	0.02 to 0.3	0.020
Cutoff One[a]					0.2	0.2	0.05 to 0.4	0.013
Cutoff Two[b]					0.4	0.5	0.1 to 0.8	0.007
Cutoff Three[c]					1.0	1.0	0.04 to 2.0	0.042

[a] A participant in the intervention arm is deemed a complier if they have reported undertaking informal mindfulness practice at least occasionally.

[b] A participant in the intervention arm is deemed a complier if they have reported undertaking informal mindfulness practice at least several times a week.

[c] A participant in the intervention arm is deemed a complier if they have reported undertaking informal mindfulness practice daily.

Supplementary Table S8: Instrumental Variable Analysis of the teacher outcomes following personal MT (MBCT-L) and SBMT teacher training, but before delivery of the pupil MT, with allocated group as an instrument for formal mindfulness practice

**Outcome**	**SBMT arm (I)**		**TAU arm (C)**		**Unadjusted mean Difference (I-C)**	**Adjusted mean difference (I-C)**		
	**N**	**mean (SD)**	**N**	**mean (SD)**	**estimate**	**estimate**	**95% CI**	**p-value**
**Well-being – Maslach Burn-Out Inventory (MBI)**								
Exhaustion								
ITT	264	2.5 (1.2)	221	2.5 (1.3)	-0.1	-0.2	-0.3 to 0.01	0.066
Cutoff One[a]					-0.2	-0.2	-0.5 to -0.01	0.038
Cutoff Two[b]					-0.4	-0.4	-0.9 to 0.1	0.124
Cutoff Three[c]					-2.0	-1.5	-4.0 to 0.9	0.224
Depersonalisation								
ITT	264	0.7 (0.8)	221	0.8 (1.0)	-0.1	-0.1	-0.2 to 0.1	0.367
Cutoff One[a]					-0.1	-0.1	-0.3 to 0.1	0.311
Cutoff Two[b]					-0.4	-0.2	-0.6 to 0.3	0.419
Cutoff Three[c]					-1.7	-0.4	-2.5 to 1.6	0.693
Personal Accomplishment								
ITT	264	1.0 (0.7)	221	1.1 (0.8)	-0.1	-0.1	-0.2 to 0.03	0.159

Cutoff One[a]					-0.1	-0.1	-0.3 to 0.03	0.105
Cutoff Two[b]					-0.4	-0.1	-0.4 to 0.3	0.679
Cutoff Three[c]					-1.8	-0.5	-2.2 to 1.3	0.596
**Self-efficacy Questionnaire (TSES)**								
Student Engagement Subscale								
ITT	258	7.0 (1.0)	215	6.7 (1.2)	0.3	0.2	0.04 to 0.4	0.017
Cutoff One[a]					0.4	0.3	0.1 to 0.6	0.011
Cutoff Two[b]					1.0	0.6	0.002 to 1.2	0.049
Cutoff Three[c]					4.7	2.8	-0.1 to 5.7	0.059
Instructional Practice Subscale								
ITT	258	7.4 (0.9)	215	7.4 (1.0)	-0.01	0.02	-0.2 to 0.2	0.792
Cutoff One[a]					-0.01	0.05	-0.2 to 0.3	0.696
Cutoff Two[b]					-0.03	0.05	-0.5 to 0.5	0.854
Cutoff Three[c]					-0.2	0.4	-1.8 to 2.7	0.701
Classroom Management Subscale								
ITT	258	7.7 (0.8)	215	7.6 (1.0)	0.2	0.1	-0.04 to 0.2	0.143
Cutoff One[a]					0.2	0.2	-0.05 to 0.3	0.135
Cutoff Two[b]					0.5	0.1	-0.3 to 0.6	0.539
Cutoff Three[c]					2.5	0.9	-1.3 to 3.0	0.441

**Mindfulness (FFMQ-SF)**								
ITT	260	84.7 (12.6)	215	85.4 (13.1)	-1.2	-0.4	-2.3 to 1.6	0.706
Cutoff One[a]					-1.8	-0.3	-2.9 to 2.2	0.795
Cutoff Two[b]					-4.6	0.4	-5.6 to 6.4	0.901
Cutoff Three[c]					-21.6	0.2	-26.1 to 26.5	0.989
**Mindfulness (MTS) - Interpersonal**								
ITT	262	20.7 (2.4)	216	20.7 (2.7)	-0.1	-0.1	-0.5 to 0.3	0.726
Cutoff One[a]					-0.1	-0.1	-0.6 to 0.5	0.836
Cutoff Two[b]					-0.3	-0.5	-1.9 to 0.8	0.442
Cutoff Three[c]					-1.5	-1.7	-7.6 to 4.3	0.588
**Mindfulness (MTS) - Intrapersonal**								
ITT	262	32.0 (4.9)	217	34.0 (5.7)	-1.8	-1.4	-2.2 to -0.6	<0.001
Cutoff One[a]					-2.6	-1.9	-3.0 to -0.9	<0.001
Cutoff Two[b]					-6.6	-4.4	-7.0 to -1.8	0.001
Cutoff Three[c]					-31.4	-19.3	-33.4 to -5.3	0.007
**Stress (PSS)**								
ITT	262	16.2 (7.0)	217	15.8 (7.1)	0.2	0.4	-0.6 to 1.5	0.402
Cutoff One[a]					0.4	0.6	-0.7 to 2.0	0.347
Cutoff Two[b]					1.0	1.3	-1.9 to 4.6	0.417

Cutoff Three[c]					4.9	6.3	-7.8 to 20.3	0.379
**Depression (PHQ-9)**								
ITT	262	5.1 (3.9)	217	4.7 (4.0)	0.3	0.4	-0.2 to 1.0	0.233
Cutoff One[a]					0.4	0.5	-0.3 to 1.4	0.197
Cutoff Two[b]					1.0	1.5	-0.5 to 3.5	0.152
Cutoff Three[c]					4.8	5.3	-3.9 to 14.4	0.257
**Anxiety (GAD7)**								
ITT	261	4.3 (3.6)	216	4.4 (4.0)	-0.2	0.04	-0.6 to 0.6	0.906
Cutoff One[a]					-0.3	0.1	-0.7 to 0.9	0.826
Cutoff Two[b]					-0.7	0.5	-1.5 to 2.5	0.617
Cutoff Three[c]					-3.3	4.3	-4.8 to 13.4	0.356
School ecology/climate (SCCS)								
School leadership and involvement								
ITT	269	3.9 (0.7)	222	3.8 (0.7)	0.1	0.2	0.04 to 0.3	0.011
Cutoff One[a]					0.2	0.2	0.1 to 0.4	0.004
Cutoff Two[b]					0.5	0.6	0.2 to 0.9	0.003
Cutoff Three[c]					2.5	2.6	0.7 to 4.6	0.009
Staff attitudes								
ITT	269	4.1 (0.6)	222	4.1 (0.5)	-0.1	-0.01	-0.1 to 0.1	0.902

Cutoff One[a]					-0.1	-0.002	-0.1 to 0.1	0.976
Cutoff Two[b]					-0.2	0.05	-0.3 to 0.4	0.768
Cutoff Three[c]					-1.0	0.2	-1.2 to 1.7	0.772
Respectful Climate								
ITT	269	3.8 (0.6)	222	3.7 (0.6)	0.1	0.1	-0.01 to 0.2	0.087
Cutoff One[a]					0.1	0.1	-0.01 to 0.2	0.063
Cutoff Two[b]					0.2	0.3	-0.02 to 0.6	0.069
Cutoff Three[c]					1.2	1.3	-0.05 to 2.6	0.059

[a] A participant in the intervention arm is deemed a complier if they have reported undertaking formal mindfulness practice at least occasionally.

[b] A participant in the intervention arm is deemed a complier if they have reported undertaking formal mindfulness practice at least several times a week.

[c] A participant in the intervention arm is deemed a complier if they have reported undertaking formal mindfulness practice daily.

Supplement Table S9: Instrumental Variable Analysis of the teacher outcomes following personal MT (MBCT-L) and SBMT teacher training, but before the pupil MT, with allocated group as an instrument for informal mindfulness practice

**Outcome**	**SBMT arm (I)**		**TAU arm (C)**		**Unadjusted mean difference**	**Adjusted mean difference**		
	**N**	**mean (SD)**	**N**	**mean (SD)**	**estimate**	**estimate**	**95% CI**	**p-value**
**Well-being – Maslach Burn-Out Inventory (MBI)**								
Exhaustion								
ITT	264	2.5 (1.2)	221	2.5 (1.3)	-0.1	-0.2	-0.3 to 0.01	0.066
Cutoff One[a]					-0.1	-0.2	-0.4 to -0.01	0.037
Cutoff Two[b]					-0.2	-0.3	-0.6 to -0.001	0.049
Cutoff Three[c]					-0.6	-0.9	-1.6 to -0.1	0.026
Depersonalisation								
ITT	264	0.7 (0.8)	221	0.8 (1.0)	-0.1	-0.1	-0.2 to 0.1	0.367
Cutoff One[a]					-0.1	-0.1	-0.3 to 0.1	0.314
Cutoff Two[b]					-0.2	-0.1	-0.4 to 0.1	0.321
Cutoff Three[c]					-0.5	-0.4	-1.1 to 0.2	0.186
Personal Accomplishment								
ITT	264	1.0 (0.7)	221	1.1 (0.8)	-0.1	-0.1	-0.2 to 0.03	0.159

Cutoff One[a]					-0.1	-0.1	-0.3 to 0.03	0.108
Cutoff Two[b]					-0.2	-0.1	-0.3 to 0.1	0.199
Cutoff Three[c]					-0.5	-0.4	-1.0 to 0.1	0.113
**Self-efficacy Questionnaire (TSES)**								
Student Engagement Subscale								
ITT	258	7.0 (1.0)	215	6.7 (1.2)	0.3	0.2	0.04 to 0.4	0.017
Cutoff One[a]					0.3	0.3	0.1 to 0.5	0.010
Cutoff Two[b]					0.5	0.4	0.1 to 0.7	0.014
Cutoff Three[c]					1.4	1.1	0.2 to 1.9	0.013
Instructional Practice Subscale								
ITT	258	7.4 (0.9)	215	7.4 (1.0)	-0.01	0.02	-0.2 to 0.2	0.792
Cutoff One[a]					-0.01	0.04	-0.2 to 0.2	0.705
Cutoff Two[b]					-0.02	0.1	-0.3 to 0.4	0.725
Cutoff Three[c]					-0.04	0.2	-0.5 to 0.9	0.486
Classroom Management Subscale								
ITT	258	7.7 (0.8)	215	7.6 (1.0)	0.2	0.1	-0.04 to 0.2	0.143
Cutoff One[a]					0.2	0.1	-0.04 to 0.3	0.139
Cutoff Two[b]					0.3	0.2	-0.1 to 0.4	0.209
Cutoff Three[c]					0.7	0.6	-0.1 to 1.2	0.085

**Mindfulness (FFMQ-SF)**								
ITT	260	84.7 (12.6)	215	85.4 (13.1)	-1.2	-0.4	-2.3 to 1.6	0.706
Cutoff One[a]					-1.6	-0.2	-2.6 to 2.1	0.833
Cutoff Two[b]					-2.4	-0.3	-3.8 to 3.2	0.884
Cutoff Three[c]					-6.4	0.4	-8.2 to 9.0	0.933
**Mindfulness (MTS) - Interpersonal**								
ITT	262	20.7 (2.4)	216	20.7 (2.7)	-0.1	-0.1	-0.5 to 0.3	0.726
Cutoff One[a]					-0.1	-0.05	-0.6 to 0.5	0.847
Cutoff Two[b]					-0.2	-0.1	-0.9 to 0.6	0.736
Cutoff Three[c]					-0.4	0.1	-1.7 to 1.9	0.874
**Mindfulness (MTS) - Intrapersonal**								
ITT	262	32.0 (4.9)	217	34.0 (5.7)	-1.8	-1.4	-2.2 to -0.6	<0.001
Cutoff One[a]					-2.3	-1.7	-2.7 to -0.8	<0.001
Cutoff Two[b]					-3.5	-2.6	-4.1 to -1.1	0.001
Cutoff Three[c]					-9.2	-5.3	-9.2 to -1.3	0.009
**Stress (PSS)**								
ITT	262	16.2 (7.0)	217	15.8 (7.1)	0.2	0.4	-0.6 to 1.5	0.402
Cutoff One[a]					0.4	0.6	-0.6 to 1.8	0.343
Cutoff Two[b]					0.6	0.9	-0.9 to 2.7	0.339

Cutoff Three[c]					1.5	2.1	-2.4 to 6.7	0.357
**Depression (PHQ-9)**								
ITT	262	5.1 (3.9)	217	4.7 (4.0)	0.3	0.4	-0.2 to 1.0	0.233
Cutoff One[a]					0.4	0.5	-0.2 to 1.2	0.176
Cutoff Two[b]					0.5	0.8	-0.3 to 2.0	0.132
Cutoff Three[c]					1.4	1.6	-1.3 to 4.4	0.274
**Anxiety (GAD7)**								
ITT	261	4.3 (3.6)	216	4.4 (4.0)	-0.2	0.04	-0.6 to 0.6	0.906
Cutoff One[a]					-0.2	0.1	-0.6 to 0.8	0.810
Cutoff Two[b]					-0.4	0.3	-0.8 to 1.4	0.605
Cutoff Three[c]					-0.9	0.3	-2.5 to 3.1	0.810
School ecology/climate (SCCS)								
School leadership and involvement								
ITT	269	3.9 (0.7)	222	3.8 (0.7)	0.1	0.2	0.04 to 0.3	0.011
Cutoff One[a]					0.2	0.2	0.1 to 0.4	0.004
Cutoff Two[b]					0.3	0.3	0.1 to 0.5	0.005
Cutoff Three[c]					0.7	0.9	0.4 to 1.4	0.001
Staff attitudes								
ITT	269	4.1 (0.6)	222	4.1 (0.5)	-0.1	-0.01	-0.1 to 0.1	0.902

Cutoff One[a]					-0.1	-0.0003	-0.1 to 0.1	0.996
Cutoff Two[b]					-0.1	0.001	-0.2 to 0.2	0.996
Cutoff Three[c]					-0.3	0.1	-0.4 to 0.5	0.680
Respectful Climate								
ITT	269	3.8 (0.6)	222	3.7 (0.6)	0.1	0.1	-0.01 to 0.2	0.087
Cutoff One[a]					0.1	0.1	-0.01 to 0.2	0.065
Cutoff Two[b]					0.1	0.2	-0.02 to 0.3	0.074
Cutoff Three[c]					0.3	0.4	0.01 to 0.9	0.044

[a] A participant in the intervention arm is deemed a complier if they have reported undertaking informal mindfulness practice at least occasionally.

[b] A participant in the intervention arm is deemed a complier if they have reported undertaking informal mindfulness practice at least several times a week.

[c] A participant in the intervention arm is deemed a complier if they have reported undertaking informal mindfulness practice daily.

Supplement Table S10: Instrumental Variable Analysis of the teacher outcomes following personal MT (MBCT-L) and SBMT teacher training and delivery of the pupil MT curriculum, with allocated group as an instrument for formal mindfulness practice

**Outcome**	**SBMT arm (I)**		**TAU arm (C)**		**Unadjusted mean difference**	**Adjusted mean difference**		
	**N**	**mean (SD)**	**N**	**mean (SD)**	**estimate**	**estimate**	**95% CI**	**p-value**
**Well-being – Maslach Burn-Out Inventory (MBI)**								
Exhaustion								
ITT	206	2.3 (1.1)	181	2.5 (1.3)	-0.2	-0.3	-0.5 to -0.1	0.010
Cutoff One[a]					-0.3	-0.4	-0.7 to -0.1	0.009
Cutoff Two[b]					-1.0	-0.9	-1.7 to -0.1	0.026
Cutoff Three[c]					-3.2	-2.9	-6.0 to 0.3	0.072
Depersonalisation								
ITT	206	0.8 (0.8)	181	0.9 (1.0)	-0.2	-0.2	-0.3 to 0.01	0.074
Cutoff One[a]					-0.3	-0.2	-0.5 to -0.01	0.037
Cutoff Two[b]					-0.8	-0.7	-1.4 to 0.02	0.057
Cutoff Three[c]					-2.6	-2.2	-4.9 to 0.6	0.118
Personal Accomplishment								
ITT	206	1.0 (0.7)	181	1.1 (0.8)	-0.2	-0.2	-0.3 to -0.01	0.034

Cutoff One[a]					-0.3	-0.2	-0.4 to -0.03	0.027
Cutoff Two[b]					-0.8	-0.5	-1.1 to 0.1	0.077
Cutoff Three[c]					-2.6	-1.6	-3.5 to 0.4	0.109
**Self-efficacy Questionnaire (TSES)**								
Student Engagement Subscale								
ITT	197	6.9 (1.1)	174	6.7 (1.2)	0.1	0.05	-0.2 to 0.3	0.677
Cutoff One[a]					0.2	0.1	-0.2 to 0.4	0.671
Cutoff Two[b]					0.4	0.2	-0.7 to 1.0	0.659
Cutoff Three[c]					1.4	0.5	-2.4 to 3.3	0.740
Instructional Practice Subscale								
ITT	197	7.5 (1.0)	174	7.4 (1.1)	0.1	0.1	-0.1 to 0.3	0.258
Cutoff One[a]					0.1	0.2	-0.1 to 0.4	0.247
Cutoff Two[b]					0.3	0.5	-0.3 to 1.3	0.235
Cutoff Three[c]					1.1	1.3	-1.3 to 3.9	0.318
Classroom Management Subscale								
ITT	197	7.7 (0.9)	174	7.5 (1.1)	0.1	0.04	-0.1 to 0.2	0.662
Cutoff One[a]					0.1	0.05	-0.2 to 0.3	0.665
Cutoff Two[b]					0.3	0.1	-0.6 to 0.8	0.744
Cutoff Three[c]					1.1	0.4	-1.6 to 2.5	0.684

**Mindfulness (FFMQ-SF)**								
ITT	199	86.6 (13.6)	174	85.8 (13.7)	0.5	1.3	-0.8 to 3.5	0.230
Cutoff One[a]					0.6	2.3	-0.6 to 5.3	0.114
Cutoff Two[b]					2.0	6.5	-1.8 to 14.9	0.122
Cutoff Three[c]					6.1	19.9	-9.7 to 49.6	0.186
**Mindfulness (MTS) - Interpersonal**								
ITT	201	20.8 (2.3)	176	20.5 (2.8)	0.3	0.2	-0.2 to 0.7	0.305
Cutoff One[a]					0.4	0.3	-0.3 to 1.0	0.287
Cutoff Two[b]					1.2	1.1	-0.8 to 3.0	0.237
Cutoff Three[c]					3.8	2.1	-4.1 to 8.3	0.494
**Mindfulness (MTS) - Intrapersonal**								
ITT	200	32.1 (5.1)	176	33.8 (5.5)	-1.2	-0.8	-1.6 to 0.1	0.067
Cutoff One[a]					-1.8	-1.0	-2.2 to 0.2	0.101
Cutoff Two[b]					-5.3	-2.9	-6.4 to 0.6	0.105
Cutoff Three[c]					-16.5	-8.5	-20.9 to 3.9	0.180
**Stress (PSS)**								
ITT	201	14.6 (6.5)	175	15.5 (7.4)	-0.9	-0.8	-2.0 to 0.3	0.166
Cutoff One[a]					-1.3	-1.2	-2.8 to 0.3	0.116
Cutoff Two[b]					-3.8	-3.7	-8.2 to 0.9	0.114

Cutoff Three[c]					-12.1	-12.0	-28.4 to 4.3	0.148
**Depression (PHQ-9)**								
ITT	200	4.7 (3.8)	174	5.1 (4.6)	-0.4	-0.3	-1.0 to 0.4	0.380
Cutoff One[a]					-0.6	-0.5	-1.4 to 0.4	0.270
Cutoff Two[b]					-1.8	-1.2	-3.8 to 1.5	0.381
Cutoff Three[c]					-5.6	-5.2	-13.8 to 3.3	0.231
**Anxiety (GAD7)**								
ITT	200	4.0 (3.6)	174	4.6 (4.1)	-0.7	-0.5	-1.3 to 0.2	0.142
Cutoff One[a]					-1.0	-0.8	-1.8 to 0.2	0.111
Cutoff Two[b]					-3.0	-2.3	-5.2 to 0.7	0.134
Cutoff Three[c]					-9.4	-7.5	-17.7 to 2.7	0.149
School ecology/climate (SCCS)								
School leadership and involvement								
ITT	209	3.9 (0.7)	185	3.6 (0.8)	0.2	0.2	0.03 to 0.3	0.019
Cutoff One[a]					0.3	0.3	0.1 to 0.5	0.004
Cutoff Two[b]					0.8	0.8	0.2 to 1.5	0.006
Cutoff Three[c]					2.5	2.8	0.4 to 5.1	0.022
Staff attitudes								
ITT	209	4.1 (0.6)	185	4.0 (0.6)	0.04	0.1	-0.02 to 0.2	0.111

Cutoff One[a]					0.1	0.2	0.02 to 0.3	0.025
Cutoff Two[b]					0.2	0.5	0.1 to 1.0	0.020
Cutoff Three[c]					0.8	1.8	-0.1 to 3.6	0.058
Respectful Climate								
ITT	209	3.8 (0.5)	185	3.6 (0.6)	0.2	0.2	0.03 to 0.3	0.012
Cutoff One[a]					0.2	0.2	0.1 to 0.4	0.008
Cutoff Two[b]					0.6	0.6	0.1 to 1.1	0.013
Cutoff Three[c]					1.9	2.2	0.3 to 4.2	0.027

a A participant in the intervention arm is deemed a complier if they have reported undertaking formal mindfulness practice at least occasionally.

[b] A participant in the intervention arm is deemed a complier if they have reported undertaking formal mindfulness practice at least several times a week.

[c] A participant in the intervention arm is deemed a complier if they have reported undertaking formal mindfulness practice daily.

Supplement Table S11: Instrumental Variable Analysis of the teacher outcomes following personal MT (MBCT-L) and SBMT teacher training and delivery of the student MT curriculum, with allocated group as an instrument for informal mindfulness practice

**Outcome**	**SBMT arm (I) Compliers**		**TAU arm (C)**		**Unadjusted mean difference**	**Adjusted mean difference**		
	**N**	**mean (SD)**	**N**	**mean (SD)**	**estimate**	**estimate**	**95% CI**	**p-value**
**Well-being – Maslach Burn-Out Inventory (MBI)**								
Exhaustion								
ITT	206	2.3 (1.1)	181	2.5 (1.3)	-0.2	-0.3	-0.5 to -0.1	0.010
Cutoff One[a]					-0.3	-0.3	-0.6 to -0.1	0.008
Cutoff Two[b]					-0.5	-0.5	-0.9 to -0.1	0.008
Cutoff Three[c]					-1.0	-1.1	-2.0 to -0.2	0.016
Depersonalisation								
ITT	206	0.8 (0.8)	181	0.9 (1.0)	-0.2	-0.2	-0.3 to 0.01	0.074
Cutoff One[a]					-0.2	-0.2	-0.4 to -0.01	0.041
Cutoff Two[b]					-0.4	-0.3	-0.7 to -0.03	0.032
Cutoff Three[c]					-0.8	-0.8	-1.5 to -0.02	0.045
Personal Accomplishment								
ITT	206	1.0 (0.7)	181	1.1 (0.8)	-0.2	-0.2	-0.3 to -0.01	0.034

Cutoff One[a]					-0.2	-0.2	-0.4 to -0.03	0.025
Cutoff Two[b]					-0.4	-0.3	-0.6 to -0.1	0.019
Cutoff Three[c]					-0.8	-0.6	-1.2 to -0.03	0.038
**Self-efficacy Questionnaire (TSES)**								
Student Engagement Subscale								
ITT	197	6.9 (1.1)	174	6.7 (1.2)	0.1	0.05	-0.2 to 0.3	0.677
Cutoff One[a]					0.1	0.1	-0.2 to 0.3	0.651
Cutoff Two[b]					0.2	0.1	-0.3 to 0.5	0.570
Cutoff Three[c]					0.5	0.2	-0.7 to 1.1	0.645
Instructional Practice Subscale								
ITT	197	7.5 (1.0)	174	7.4 (1.1)	0.1	0.1	-0.1 to 0.3	0.258
Cutoff One[a]					0.1	0.1	-0.1 to 0.4	0.253
Cutoff Two[b]					0.2	0.2	-0.1 to 0.6	0.204
Cutoff Three[c]					0.3	0.5	-0.3 to 1.2	0.217
Classroom Management Subscale								
ITT	197	7.7 (0.9)	174	7.5 (1.1)	0.1	0.04	-0.1 to 0.2	0.662
Cutoff One[a]					0.1	0.04	-0.2 to 0.2	0.673
Cutoff Two[b]					0.2	0.1	-0.2 to 0.4	0.599
Cutoff Three[c]					0.3	0.2	-0.5 to 0.8	0.647

**Mindfulness (FFMQ-SF)**								
ITT	199	86.6 (13.6)	174	85.8 (13.7)	0.5	1.3	-0.8 to 3.5	0.230
Cutoff One[a]					0.6	1.9	-0.7 to 4.4	0.152
Cutoff Two[b]					0.9	3.2	-0.7 to 7.1	0.105
Cutoff Three[c]					2.0	6.5	-1.7 to 14.6	0.117
**Mindfulness (MTS) - Interpersonal**								
ITT	201	20.8 (2.3)	176	20.5 (2.8)	0.3	0.2	-0.2 to 0.7	0.305
Cutoff One[a]					0.3	0.3	-0.2 to 0.9	0.244
Cutoff Two[b]					0.5	0.6	-0.3 to 1.6	0.167
Cutoff Three[c]					1.2	1.1	-0.8 to 3.1	0.249
**Mindfulness (MTS) - Intrapersonal**								
ITT	200	32.1 (5.1)	176	33.8 (5.5)	-1.2	-0.8	-1.6 to 0.1	0.067
Cutoff One[a]					-1.5	-0.9	-1.9 to 0.1	0.083
Cutoff Two[b]					-2.4	-1.3	-2.8 to 0.3	0.121
Cutoff Three[c]					-5.3	-2.6	-6.1 to 0.8	0.134
**Stress (PSS)**								
ITT	201	14.6 (6.5)	175	15.5 (7.4)	-0.9	-0.8	-2.0 to 0.3	0.166
Cutoff One[a]					-1.1	-1.0	-2.4 to 0.3	0.138
Cutoff Two[b]					-1.7	-1.6	-3.7 to 0.5	0.131

Cutoff Three[c]					-3.8	-3.6	-8.1 to 0.9	0.117
**Depression (PHQ-9)**								
ITT	200	4.7 (3.8)	174	5.1 (4.6)	-0.4	-0.3	-1.0 to 0.4	0.380
Cutoff One[a]					-0.5	-0.4	-1.2 to 0.4	0.328
Cutoff Two[b]					-0.8	-0.5	-1.7 to 0.7	0.377
Cutoff Three[c]					-1.8	-1.2	-3.9 to 1.5	0.366
**Anxiety (GAD7)**								
ITT	200	4.0 (3.6)	174	4.6 (4.1)	-0.7	-0.5	-1.3 to 0.2	0.142
Cutoff One[a]					-0.8	-0.7	-1.5 to 0.2	0.114
Cutoff Two[b]					-1.3	-1.1	-2.4 to 0.3	0.120
Cutoff Three[c]					-3.0	-2.3	-5.2 to 0.6	0.115
School ecology/climate (SCCS)								
School leadership and involvement								
ITT	209	3.9 (0.7)	185	3.6 (0.8)	0.2	0.2	0.03 to 0.3	0.019
Cutoff One[a]					0.2	0.2	0.1 to 0.4	0.007
Cutoff Two[b]					0.3	0.4	0.1 to 0.7	0.006
Cutoff Three[c]					0.8	0.9	0.2 to 1.5	0.009
Staff attitudes								
ITT	209	4.1 (0.6)	185	4.0 (0.6)	0.04	0.1	-0.02 to 0.2	0.111

Cutoff One[a]					0.1	0.1	0.01 to 0.3	0.039
Cutoff Two[b]					0.1	0.2	0.01 to 0.4	0.038
Cutoff Three[c]					0.2	0.5	0.1 to 1.0	0.021
Respectful Climate								
ITT	209	3.8 (0.5)	185	3.6 (0.6)	0.2	0.2	0.03 to 0.3	0.012
Cutoff One[a]					0.2	0.2	0.05 to 0.3	0.009
Cutoff Two[b]					0.3	0.3	0.1 to 0.5	0.013
Cutoff Three[c]					0.6	0.6	0.1 to 1.1	0.012

[a] A participant in the intervention arm is deemed a complier if they have reported undertaking informal mindfulness practice at least occasionally.

[b] A participant in the intervention arm is deemed a complier if they have reported undertaking informal mindfulness practice at least several times a week.

[c] A participant in the intervention arm is deemed a complier if they have reported undertaking informal mindfulness practice daily.

Supplement Table S12: Main comparisons of teacher outcomes at post-personal MT (MBCT-L) and SBMT teacher training : complete-case analysis

**Outcome**	**SBMT arm (I)**		**TAU arm (C)**		**Unadjusted**	**Adjusted mean difference (I-C)**			**ICC[a]**
	**N**	**mean (SD)**	**N**	**mean (SD)**	**mean difference (I-C)**	**estimate**	**95% CI**	**p-value**	
Burnout – Maslach Burn-Out Inventory (MBI)									
Emotional Exhaustion	264	2.5 (1.2)	221	2.5 (1.3)	-0.05	-0.1	-0.3 to 0.04	0.126	0.000
Depersonalization	264	0.7 (0.8)	221	0.8 (1.0)	-0.1	-0.1	-0.2 to 0.1	0.278	0.150
Personal Accomplishment	264	1.0 (0.7)	221	1.1 (0.8)	-0.1	-0.1	-0.2 to -0.01	0.027	0.026
Self-efficacy Questionnaire (TSES)									
Student Engagement subscale	258	7.0 (1.0)	215	6.7 (1.2)	0.3	0.2	0.04 to 0.3	0.016	0.084
Instructional Practice subscale	258	7.4 (0.9)	215	7.4 (1.0)	0.01	0.04	-0.1 to 0.2	0.603	0.078
Classroom Management subscale	258	7.7 (0.8)	215	7.6 (1.0)	0.2	0.1	-0.01 to 0.3	0.074	0.055
Mindfulness (FFMQ-SF)	260	84.7 (12.6)	215	85.4 (13.1)	-0.4	0.07	-2.0 to 2.1	0.946	0.059
Mindfulness in Teaching (MTS)									
Interpersonal	262	20.7 (2.4)	216	20.7 (2.7)	0.02	0.06	-0.4 to 0.5	0.785	0.028
Intrapersonal	262	32.0 (4.9)	217	34.0 (5.7)	-2.0	-1.5	-2.3 to -0.7	<0.001	0.061
Perceived Stress (PSS)	262	16.2 (7.0)	217	15.8 (7.1)	0.3	0.3	-0.6 to 1.3	0.508	0.009
Depression (PHQ-9)	262	5.1 (3.9)	217	4.7 (4.0)	0.3	0.4	-0.2 to 1.0	0.249	0.000
Anxiety (GAD7)	261	4.3 (3.6)	216	4.4 (4.0)	-0.02	0.09	-0.5 to 0.7	0.757	0.000

School ecology/climate (SCCS)									
School Leadership and Involvement	269	3.9 (0.7)	222	3.8 (0.7)	0.2	0.2	0.1 to 0.3	0.004	0.196
Staff Attitudes	269	4.1 (0.6)	222	4.1 (0.5)	-0.02	0.01	-0.1 to 0.1	0.837	0.226
Respectful Climate	269	3.8 (0.6)	222	3.7 (0.6)	0.1	0.1	-0.01 to 0.2	0.075	0.269

[a] Intra-cluster (intra-school) correlation coefficients (ICCs) from crude (unadjusted) analyses.

Supplement Table S13: Main comparisons of teacher outcomes at post-personal MT (MBCT-L) and SBMT teacher training: complete-case analysis

**Outcome**	**SBMT arm (I)**		**TAU arm (C)**		**Unadjusted**	**Adjusted mean difference (I-C)**			**ICC[a]**
	**N**	**mean (SD)**	**N**	**mean (SD)**	**mean difference (I-C)**	**estimate**	**95% CI**	**p-value**	
Burnout – Maslach Burn-Out Inventory (MBI)									
Emotional Exhaustion	206	2.3 (1.1)	181	2.5 (1.3)	-0.2	-0.3	-0.5 to -0.1	0.001	0.017
Depersonalization	206	0.8 (0.8)	181	0.9 (1.0)	-0.2	-0.2	-0.3 to 0.001	0.051	0.065
Personal Accomplishment	206	1.0 (0.7)	181	1.1 (0.8)	-0.1	-0.2	-0.3 to -0.1	0.004	0.000
Self-efficacy Questionnaire (TSES)									
Student Engagement subscale	197	6.9 (1.1)	174	6.7 (1.2)	0.2	0.1	-0.1 to 0.3	0.275	0.140
Instructional Practice subscale	197	7.5 (1.0)	174	7.4 (1.1)	0.1	0.2	-0.01 to 0.4	0.065	0.097
Classroom Management subscale	197	7.7 (0.9)	174	7.5 (1.1)	0.2	0.1	-0.04 to 0.3	0.130	0.073
Mindfulness (FFMQ-SF)	199	86.6 (13.6)	174	85.8 (13.7)	0.8	2.3	0.1 to 4.4	0.041	0.014
Mindfulness in Teaching (MTS)									
Interpersonal	201	20.8 (2.3)	176	20.5 (2.8)	0.3	0.3	-0.1 to 0.8	0.172	0.039
Intrapersonal	200	32.1 (5.1)	176	33.8 (5.5)	-1.7	-0.8	-1.8 to 0.1	0.090	0.062
Perceived Stress (PSS)	201	14.6 (6.5)	175	15.5 (7.4)	-0.9	-1.3	-2.6 to -0.1	0.041	0.031
Depression (PHQ-9)	200	4.7 (3.8)	174	5.1 (4.6)	-0.4	-0.5	-1.2 to 0.3	0.222	0.000
Anxiety (GAD7)	200	4.0 (3.6)	174	4.6 (4.1)	-0.6	-0.7	-1.4 to 0.1	0.084	0.000

School ecology/climate (SCCS)										
School Leadership and Involvement	209	3.9 (0.7)	185	3.6 (0.8)	0.3	0.3	0.1 to 0.4	<0.001	0.204
Staff Attitudes	209	4.1 (0.6)	185	4.0 (0.6)	0.1	0.1	0.03 to 0.2	0.014	0.241
Respectful Climate	209	3.8 (0.5)	185	3.6 (0.6)	0.2	0.2	0.1 to 0.3	0.001	0.323

[a] Intra-cluster (intra-school) correlation coefficients (ICCs) from crude (unadjusted) analyses.

Supplement Table S14: Main comparisons of teacher outcomes at 1-year follow-up: complete-case analysis

**Outcome**	**SBMT arm (I)**		**TAU arm (C)**		**Unadjusted**	**Adjusted mean difference (I-C)**			**ICC[a]**
	**N**	**mean (SD)**	**N**	**mean (SD)**	**mean difference (I-C)**	**estimate**	**95% CI**	**p-value**	
Burnout – Maslach Burn-Out Inventory (MBI)									
Emotional Exhaustion	210	2.4 (1.2)	171	2.5 (1.2)	-0.1	-0.1	-0.3 to 0.1	0.233	0.053
Depersonalization	210	0.7 (0.7)	171	0.9 (0.9)	-0.1	-0.1	-0.2 to 0.1	0.287	0.102
Personal Accomplishment	210	1.0 (0.7)	171	1.1 (0.8)	-0.1	-0.1	-0.3 to -0.01	0.042	0.000
Self-efficacy Questionnaire (TSES)									
Student Engagement subscale	204	7.0 (1.1)	162	6.8 (1.1)	0.2	0.2	-0.02 to 0.4	0.073	0.141
Instructional Practice subscale	204	7.5 (0.8)	162	7.5 (1.0)	0.03	0.05	-0.1 to 0.2	0.556	0.098
Classroom Management subscale	204	7.8 (0.8)	162	7.6 (0.9)	0.2	0.1	-0.04 to 0.3	0.132	0.055
Mindfulness (FFMQ-SF)	205	85.4 (12.8)	162	86.1 (13.9)	-0.6	0.5	-1.8 to 2.7	0.689	0.036
Mindfulness in Teaching (MTS)									
Interpersonal	208	20.8 (2.7)	168	20.9 (2.4)	-0.1	-0.1	-0.5 to 0.4	0.731	0.000
Intrapersonal	208	32.1 (5.0)	168	33.4 (5.6)	-1.4	-0.7	-1.7 to 0.2	0.132	0.123
Perceived Stress (PSS)	207	15.3 (6.9)	165	15.9 (7.4)	-0.6	-0.8	-2.1 to 0.4	0.193	0.057
Depression (PHQ-9)	206	4.7 (4.1)	164	5.0 (4.2)	-0.4	-0.2	-1.0 to 0.6	0.624	0.039
Anxiety (GAD7)	206	4.2 (3.9)	163	4.3 (4.1)	-0.1	-0.03	-0.8 to 0.7	0.947	0.024

School ecology/climate (SCCS)									
School Leadership and Involvement	211	3.9 (0.7)	171	3.8 (0.7)	0.1	0.1	-0.1 to 0.3	0.282	0.298
Staff Attitudes	211	4.1 (0.6)	171	4.1 (0.5)	0.04	0.08	-0.04 to 0.2	0.199	0.293
Respectful Climate	211	3.9 (0.5)	171	3.7 (0.6)	0.1	0.1	0.01 to 0.3	0.035	0.267

[a] Intra-cluster (intra-school) correlation coefficients (ICCs) from crude (unadjusted) analyses.

References

1. Department of Education. Personal, social, health and economic (PSHE) education: A mapping study of the prevalent models of delivery and their effectiveness. London; 2010. Contract No.: DFE-RR080.
2. Kuyken W, Nuthall E, Byford S et al. The effectiveness and cost-effectiveness of a mindfulness training programme in schools compared with normal school provision (MYRIAD): study protocol for a randomised controlled trial. Trials. 2017;18(1):194.
3. Montero-Marin J, Nuthall, E, Byford, S et al. Update to the effectiveness and cost-effectiveness of a mindfulness training programme in schools compared with normal school provision (MYRIAD): study protocol for a randomised controlled trial. Trials. 2021;22(1):254.
4. Juneau AK. School climate and connectedness survey [measurement instrument]. Association of Alaska School Boards; 2015.
5. Maslach C, Jackson SE, Leiter MP. Maslach burnout inventory manual, third edition. Palo Alto, California: Press CP 1996.
6. Byrne, B.M. The Maslach Burnout Inventory: Testing for factorial validity and invariance across elementary, intermediate, and secondary teachers. *Journal of Occupational and Organizational Psychology* 1993;66:197-212.
7. Kokkinos, CM. Factor Structure and Psychometric Properties of the Maslach Burnout Inventory-Educators Survey among Elementary and Secondary School Teachers in Cyprus. *Stress and Health* 2006;22,25-33.
8. Frank JL, Jennings PA, Greenberg MT. Validation of the Mindfulness in Teaching Scale. *Mindfulness* 2016;**7**:155-63.
9. Cohen S, Kamarck T, Mermelstein R. A Global Measure of Perceived Stress. *Journal of Health and Social Behavior* 1983;24:385-96.
10. Kroenke K, Spitzer RL. The PHQ-9: A new depression diagnostic and severity measure. *Psychiatric Annals* 2002;32(9):509–515.
11. Kroenke K, Spitzer RL, Williams JB. The PHQ-9: validity of a brief depression severity measure. *J Gen Intern Med* 2001;16:606-13.
12. Spitzer RL, Kroenke K, Williams JBW, et al. A brief measure for assessing generalized anxiety disorder - The GAD-7. *Arch Intern Med* 2006;166:1092-7.
13. Löwe B, Decker O, Müller S, et al. Validation and standardization of the generalized anxiety disorder screener (GAD-7) in the general population. *Med Care* 2008;46:266–274.
14. Bohlmeijer E, ten Klooster PM, Fledderus M, et al. Psychometric properties of the five facet mindfulness questionnaire in depressed adults and development of a short form. *Assessment* 2011;18(3):308-20.
15. Baer RA, Smith GT, Hopkins J, et al. (2006). Using self-report assessment methods to explore facets of mindfulness. *Assessment* 2006;13:27-45.
16. Baer RA, Smith GT, Lykins E, et al. (2008). Construct validity of the five-facet mindfulness questionnaire in meditating and non-meditating samples. *Assessment* 2008;15:329–342.
17. Tschannen-Moran M, Woolfolk HA. Teacher efficacy: Capturing an elusive construct. *Teach Teach Educ* 2001;17:783-805.
18. Fives HR, Buehl MM. Examining the Factor Structure of the Teachers' Sense of Efficacy Scale, *The Journal of Experimental Education* 2009;78:118-134.
19. Department of Education. Schools, pupils and their characteristics: January 2020 London, UK2020 [Available from: https://www.gov.uk/government/statistics/schools-pupils-and-their-characteristics-january-2020].
